# miR156-*SPL* modules regulate induction of somatic embryogenesis in citrus callus

**DOI:** 10.1093/jxb/ery132

**Published:** 2018-04-05

**Authors:** Jian-Mei Long, Chao-Yang Liu, Meng-Qi Feng, Yun Liu, Xiao-Meng Wu, Wen-Wu Guo

**Affiliations:** Key Laboratory of Horticultural Plant Biology (Ministry of Education), Huazhong Agricultural University, Wuhan, China

**Keywords:** Callus, citrus, knock-down, miR156, overexpression, somatic embryogenesis, SPL

## Abstract

miR156 is a highly conserved plant miRNA and has been extensively studied because of its versatile roles in plant development. Here, we report a novel role of miR156 in regulating somatic embryogenesis (SE) in citrus, one of the most widely cultivated fruit crops in the world. SE is an important means of *in vitro* regeneration, but over the course of long-term sub-culturing there is always a decline in the SE potential of the preserved citrus embryogenic callus, and this represents a key obstacle for citrus biotechnology. In this study, the SE competence of citrus callus of wild kumquat (*Fortunella hindsii*) was significantly enhanced by either overexpression of csi-miR156a or by individual knock-down of the two target genes, *CsSPL3* and *CsSPL14*, indicating that the effect of miR156-*SPL* modules was established during the initial phases of SE induction. Biological processes that might promote SE in response to miR156 overexpression were explored using RNA-seq, and mainly included hormone signaling pathways, stress responses, DNA methylation, and the cell cycle. CsAKIN10 was identified as interacting protein of CsSPL14. Our results provide insights into the regulatory pathway through which miR156-*SPL* modules enhance the SE potential of citrus callus, and provide a theoretical basis for improvement of plant SE competence.

## Teaser

Overexpression of csi-miR156a enhances the somatic embryogenesis capability of citrus through downstream repression of *CsSPL3* and *CsSPL14*.

## Introduction

Plant cells possess the capacity to regenerate complete plants through somatic embryogenesis (SE) and *de novo* organogenesis *in vitro*, a phenomenon known as cell totipotency (Ikeuchi *et al.*, 2016; Zhang *et al.*, 2017). *De novo* organogenesis is the process by which shoots or roots are originated from explants either directly or indirectly (with callus formation step) (Liu *et al.*, 2014; Pulianmackal *et al.*, 2014), whereas SE refers to the process by which somatic cells first de-differentiate and then differentiate into embryonic cells that generate embryos and subsequently produce intact plants (Verdeil *et al.*, 2007). SE is an effective and powerful means of *in vitro* regeneration for species that have a long reproductive cycle or a low seed set (Smertenko and Bozhkov, 2014). As a classic example of plant cell totipotency, SE has long attracted attention and has been widely studied in many species. Following the first report of SE in carrot (Steward *et al.*, 1958), initial studies focused on manipulation to induce somatic embryos in various other plant species, whereas in more recent years substantial efforts have been made to uncover the regulatory mechanisms of SE. Stress and/or hormones are known to be common induction factors that initiate somatic embryos (Fehér, 2015). Auxin and cytokinin have often been used for promoting somatic embryo formation, as well as heavy metal ions, and osmotic and dehydration stress (Jiménez, 2005; Zavattieri *et al.*, 2010). A number of genes have been studied in Arabidopsis, and in particular critical transcription factors (TFs) that regulate SE have been profiled and their functions investigated (Gliwicka *et al.*, 2013). Four main types of TFs that positively regulate SE in Arabidopsis have been considered in particular, namely *LEAFY COTYLEDON* (*LEC*) genes (including *LEC1*, *LEC2*, and *FUSCA3*, *FUS3*), *AGAMOUS-LIKE15* (*AGL15*), *BABY BOOM* (*BBM*), and *WUSCHEL RELATED HOMEOBOX* (*WOX*) (Guan *et al.*, 2016). In addition, a model for crosstalk among hormones, stress, and TFs for SE induction in Arabidopsis has been proposed (Germana and Lambardi, 2016), in which stress and exogenous hormones, two major SE inducers, trigger *de novo* synthesis and signaling of endogenous hormones, with subsequent activation of a TF-related regulatory network. To date, however, in-depth investigation of the post-transcriptional regulation of SE has been lacking.

miRNAs, which are endogenous single-strand non-coding RNAs of 20–24 nucleotides (nt) in length, are known to regulate gene expression at the post-transcriptional level through cleavage of mRNAs and/or translational repression of the corresponding target genes. miRNAs have been found to regulate a large number of biological processes in plants, including growth, development, metabolism, and abiotic and biotic stress responses (reviewed in Xie *et al.*, 2015; Li and Zhang, 2016). miRNAs involved in SE have been identified in different plants including both annuals and perennials, such as rice (Luo *et al.*, 2006; Chen *et al.*, 2011), maize (Chávez-Hernández *et al.*, 2015), cotton (Yang *et al.*, 2013), citrus (Wu *et al.*, 2015), longan (Lin and Lai, 2013), and larch (Zhang *et al.*, 2012). Among the SE-related miRNAs that have been identified, miR156 has been reported to be involved in the rice callus phase transition from the undifferentiated to the differentiated state (Luo *et al.*, 2006), and it is required for early zygotic embryogenesis (Nodine and Bartel, 2010; Willmann *et al.*, 2011). Because of its abundant accumulation specifically in embryonic calluses and throughout the SE induction process in citrus (Wu *et al.*, 2015), miR156 may have a role in mediating the regulation of SE.

miR156 is one of the most conserved miRNA families in plants, and it targets *SQUAMOSA PROMOTER BINDING PROTEIN-LIKE* (*SPL*) TF genes (Rhoades *et al.*, 2002). miR156-*SPL* modules have been reported to regulate multiple biological processes, including juvenile-to-adult phase transition, development of leaves, roots and fruit, fertility, stress responses, and secondary metabolism (Wu and Poethig, 2006; Wang *et al.*, 2009; Xing *et al.*, 2010; Gou *et al.*, 2011; Cui *et al.*, 2014; Rubio-Somoza *et al.*, 2014; Silva *et al.*, 2014; Yu *et al.*, 2015*a*, 2015*b*). The miR156-mediated regulation of phase change is conserved among angiosperms, with overexpression of miR156 extending the juvenile phase and delaying flowering, whereas inactivation of miR156 accelerates the transition (reviewed in Wang, 2014; Wang and Wang, 2015). Since it has high abundance in seedlings and gradually declines with the progression of development, miR156 has been proposed to be a common age marker for most plants, including the woody perennial poplar (Wang *et al.*, 2011; Wang, 2014). In addition to its well-documented functions in plant systems *in vivo*, miR156 has also been shown to act as an intrinsic regulator of *in vitro* shoot regeneration (Zhang *et al.*, 2015), a mode of regeneration that is different from SE. miR156 and *SPL* TFs have also been identified in fruit trees. Fifteen of the 27 *SPL* genes in apple and 10 of the 15 *SPL*s in citrus have been identified as putative targets of miR156 (Li *et al.*, 2013; Shalom *et al.*, 2015; Liu *et al.*, 2017). Ectopic expression of apple *Md-miR156h* in Arabidopsis causes an extended juvenile phase and abnormal fruit set (Sun *et al.*, 2013), whereas overexpression of citrus *CiSPL5* promotes flowering in Arabidopsis (Shalom *et al.*, 2015). However, miR156-mediated regulation of SE has rarely been reported in either annual or perennial plants, except for a recent report concerning the regulatory roles of GhmiR157a/GhSPL10 in initial cellular de-differentiation and callus proliferation in cotton (Wang *et al.*, 2018).

Citrus is one of the most widely cultivated fruit crops in the world. Biotechnology provides a promising approach for citrus genetic improvement (Guo *et al.*, 2007*a*, 2007*b*) and can circumvent the obstacles presented to conventional breeding, such as long juvenility, nucellar polyembryony, pollen and/or ovule sterility, and widespread incompatibility (Wang *et al.*, 2017). However, for the majority of citrus cultivars, the SE capability of embryogenic calluses always declines over long-term sub-culturing, limiting its efficiency as a means of regeneration. Therefore, maintenance and enhancement of SE capability is of great importance for citrus improvement. SE-related genes, proteins, and small RNAs have been profiled previously (Pan *et al.*, 2009; Ge *et al.*, 2012; Wu *et al.*, 2015), but little is understood about the regulatory pathways involved. In a previous study, we found that miR156 accumulated to abundant levels in the embryogenic callus of citrus and throughout the SE process, which suggested that it has a role in SE (Wu *et al.*, 2015). In the present study, we first isolated the full-length of csi-miR156a coding gene, *CsMIR156A*. Through overexpression of csi-miR156a and suppression of miR156-targeted *SPL*s in citrus calluses, we confirmed the hypothesis that miR156-*SPL*s regulate citrus SE. In miR156 overexpression callus lines, biological processes such as hormone signaling and stress responses were significantly enhanced. CsSPL14, one of the targets acting downstream of miR156 during SE, was found to interact with the SnRK1 catalytic subunit alpha-KIN10 (CsAKIN10). Our results confirm the regulatory roles of miR156-*SPL* modules in citrus SE, thus providing new insights into miRNA-mediated regulation of plant SE.

## Materials and methods

### Plant material

The embryogenic callus (EC) used for genetic transformation was derived from wild kumquat (*Fortunella hindsii*), a precocious citrus germplasm native to China. The EC was initially derived from the hypocotyl region of seedlings cultured on MT medium (Murashige and Tucker, 1969) in darkness at 25 °C and preserved by subculture over a 20-d interval in light conditions. However, after preservation for nearly 10 years, the wild-type EC had lost the majority of its SE capability.

In order to detect and compare the expression level of genes, ECs of another five genotypes were collected, namely ‘American’ sour orange (‘AS’, *Citrus aurantium*), ‘Valencia’ sweet orange (‘V’, *C. sinensis*), ‘Newhall’ navel orange (‘NH’, *C. sinensis*), ‘Anliu’ sweet orange (‘AL’, *C. sinensis*), and ‘Guoqing No.1’ Satsuma mandarin (‘G1’, *C. unshiu*). The five ECs were all initially induced from aborted seeds, and maintained different SE capabilities after long-term preservation: AS and V maintained high SE competence, followed by NH and AL with weaker SE capability, whilst the EC of G1 was recalcitrant in forming somatic embryos.

For DNA extraction and gene amplification, leaves were collected from four genotypes representative of the different SE capacities, namely ‘V’, ‘NH’, ‘G1’, and wild kumquat, from adult trees grown at the Institute of Citrus Science, Huazhong Agricultural University.

### Gene cloning and sequence analysis

Total RNA was extracted from calluses using Trizol reagent as described previously (Liu *et al.*, 2006). According to the precursor sequence of miR156a reported in previous study (Liu *et al.*, 2017), the full-length miR156a coding gene was obtained by 5′- and 3′-rapid amplification of cDNA ends (RACE) according to the manufacturer’s protocols (Invitrogen, USA) using the primers listed in Supplementary Table S1 at *JXB* online. The PCR products were inserted into the pTOPO-TA vector and sequenced. Full-length sequences were amplified from genomic DNA of leaves from all six cultivars described above. Genomic DNA was extracted as described previously (Cheng *et al.*, 2003). Sequence alignment was performed using ClustalX (Thompson *et al.*, 2002) and GeneDoc (Nicholas *et al.*, 1997). According to the nomenclature reported previously (Liu *et al.*, 2017), protein sequences of CsSPL3 and CsSPL14 were retrieved from the sweet orange genome database (http://citrus.hzau.edu.cn/orange/download/data.php). Protein sequences of the 10 Arabidopsis SPLs targeted by miR156 (Xing *et al.*, 2010) were downloaded from TAIR (http://www.arabidopsis.org/), namely AT5G43270 (AtSPL2), AT2G33810 (AtSPL3), AT1G53160 (AtSPL4), AT3G15270 (AtSPL5), AT1G69170 (AtSPL6), AT2G42200 (AtSPL9), AT1G27370 (AtSPL10), AT1G27360 (AtSPL11), AT5G50570 (AtSPL13), and AT3G57920 (AtSPL15). In addition, sequences of 11 miR156-targetd rice SPLs (Xie *et al.*, 2006) were downloaded from http://www.ricedata.cn/gene/ with the following accession numbers: LOC_Os01g69830 (OsSPL2), LOC_Os02g04680 (OsSPL3), LOC_Os02g07780 (OsSPL4), LOC_Os04g46580 (OsSPL7), LOC_Os06g45310 (OsSPL11), LOC_Os06g49010 (OsSPL12), LOC_Os07g32170 (OsSPL13), LOC_Os08g39890 (OsSPL14), LOC_Os08g41940 (OsSPL16), LOC_Os09g31438 (OsSPL17), and LOC_Os09g33944 (OsSPL18). A phylogenetic tree was generated by MEGA6 using the neighbor-joining method (Tamura *et al.*, 2013).

### Plasmid construction and callus transformation

The plasmid pCAMBIA13011 containing the miR156 precursor MIR156a overexpression construct was generated previously (Liu *et al.*, 2017). Briefly, a 187-bp fragment of the csi-miR156 precursor was amplified by PCR and cloned into plasmid pCAMBIA13011, a derivative of pCAMBIA1301 carrying the 35S promoter and the RBS terminator, which was kindly provided by Dr Hongwu Bian of Zhejiang University. For the construction of RNAi vectors, gene-specific fragments in the 3′-untranslated region of *CsSPL3* and *CsSPL14* were amplified from wild kumquat, and cloned into the pHGRV plasmid. The inserted genes were all driven by the CaMV *35S* promoter, and transformed into a long-term preserved callus of wild kumquat. The primers used for vector constructions are listed in Supplementary Table S1. Callus transformation was performed according to Duan *et al.* (2007). The independent transformed callus lines were recovered from tiny pieces of antibiotic-resistant callus, as described previously (Cao *et al.*, 2012). The wild-type and transgenic calluses were preserved by periodical sub-culturing on MT medium at 20-d intervals.

### GUS staining and transmission electronic microscopy analysis

GUS staining assay was conducted as described previously (Huang *et al.*, 2017). Calluses were submerged in X-Gluc solution, vacuumed, and incubated at 37 °C, then destained in 70% ethanol until the negative control callus turned white. The calluses were photographed under a stereoscopic microscope (Leica, Germany).

Transmission electronic microscope (TEM) analysis of callus was carried out according to Cao *et al.* (2012). After a new round of 20-d subculture, the fresh callus samples were collected and fixed in 2.5% glutaraldehyde, and then transferred into 0.1 M phosphate buffer with 2% OsO_4_. After dehydration, the calluses were embedded in epoxy resin and SPI-812. Ultrathin sections were obtained using an ultramicrotome (UC61, Leica) and then stained with uranyl acetate and lead citrate. Images were photographed using a HITACHI H-7650 TEM.

### Induction of somatic embryos and comparisons of SE capability

To obtain homogenized callus cells, callus cultures were transferred into liquid MT medium with 0.5 g l^–1^ malt extract and 1.5 g l^–1^ glutamine and placed on a shaker at 120 rpm for 2 weeks. To induce somatic embryos, calluses were then transferred into MT solid medium supplemented with 2% glycerol instead of sucrose. When somatic embryos were visible, samples of 0.3–0.5 g fresh callus were collected and the visible embryos were counted under a stereoscopic microscope. Three bottles of culture formed the bio-replicates, whilst three counts were conducted for each bottle as the technical replicates. The SE capability was calculated as the number of somatic embryos formed per gram of fresh callus.

### Comparison of gene expression levels

Both wild-type and the transformed calluses of wild kumquat were collected at 0, 20, 40 and 60 d after induction (DAI) of somatic embryos, and snap-frozen in liquid nitrogen, and stored at –80°C. Total RNA from the calluses was extracted using RNAiso Plus (Takara, Dalian, China) according to the manufacturer’s instructions, followed by quality evaluation using Bioanalyzer 2100 (Agilent, Germany).

To compare gene expression profiles, duplicate biological replicates of the csi-miR156a-overexpressed (OE) lines and the wild-type (WT) were used. Specifically, two independent csi-miR156a OE lines, OE-L13 and OE-L22, were selected and considered as two biological replicates. At 40 DAI, the color of transgenic calluses became dark yellow, whereas the WT callus was still light yellow, similar to that of calluses without induction. In addition, somatic embryos had emerged at 60 DAI in the OE lines, and hence RNA-seq libraries were constructed for the OE lines and the WT at 40 and 60 DAI (i.e. at the pre-SE and the SE stage, respectively), using the NEBNext® Ultra™ Directional RNA Library Prep Kit for Illumina® (NEB, USA) according to the manufacturer’s instructions. The libraries were clustered on a cBot Cluster Generation System using the TruSeq SR Cluster Kit v3-cBot-HS (Illumina), and subsequently sequenced on an Illumina Hiseq 2000 platform (Novogene, Beijing) to generate 125-bp pair-end reads. After the removal of low-quality reads and contaminants, the adaptors were trimmed and the clean and high-quality reads were mapped to the genome of clementine (version10.0, https://www.citrusgenomedb.org/) using HISAT2 with two mismatches allowed. The aligned reads were then assembled using StringTie (Pertea *et al.*, 2016). The correlations between the two biological replicates were measured using the cor function in R (www.r-project.org) using transcript abundance calculated as fragments per kilobase of exon per million fragments mapped (FPKM). Raw read counts of each gene were calculated by HTSeq (Anders *et al.*, 2015), followed by identification of differentially expressed genes (DEGs) using edgeR (Robinson *et al.*, 2010), with log_2_-fold change ≥1.0 and false discovery rate (FDR) <0.001. The DEGs were aligned to the Arabidopsis protein database using blastx with *E*-value ≤10^–5^ to obtain the corresponding TAIR10 protein ID. Gene ontology (GO) functional annotations were conducted using agriGO tools (Du *et al.*, 2010), with FDR <0.05 as the cut-off to identify over-represented biological processes. The RNA-seq data was submitted to the NCBI Gene Expression Omnibus (GEO) under the accession number GSE98687.

For quantitative RT-PCR (qRT-PCR) of mRNA, RNA was reverse-transcribed into cDNA using the primeScript RT reagent Kit with gDNA eraser (Takara, Dalian, China), with *CsUBL5* as the internal control (Liu *et al.*, 2013). The primers for *CsSPL* genes have been listed in a previous study (Liu *et al.*, 2017). Stem-loop qRT-PCR for mature miRNA was conducted as described previously (Varkonyi-Gasic *et al.*, 2007), using primers listed in a previous study (Wu *et al.*, 2011), with U6 as the endogenous reference (Kou *et al.*, 2012). The qRT-PCR was conducted on an ABI Prism 7900HT (Applied Biosystems) using the SYBR Green system, with no less than two independent biological replicates, each comprising three technical replicates.

### Subcellular localization and transactivation activity assay of CsSPLs

The coding sequence regions of *CsSPL3* and *CsSPL14* were amplified and inserted into the pM999-35S vector to generate the *35S::CsSPLs-GFP* fusion constructs, whilst the full-length sequence of the interacting protein CsAKIN10 was cloned into the p2GWY7 vector to produce the *35S::CsAKIN10-YFP* (yellow fluorescent protein) fusion construct. The nuclear localization of rice OsGhd7 (Grain number, plant height, heading date-7) fused with CFP (cyan fluorescent protein) was used as a positive nucleus marker to generate the *35S::*OsGhd7-CFP construct (Xue *et al.*, 2008; Fang *et al.*, 2014). Arabidopsis protoplasts were prepared, followed by co-transformation of fusion constructs of targets and markers, as described by Wu *et al.* (2009a). The transformed protoplasts were visualized using a confocal laser-scanning microscope (Leica TCS SP8, Germany).

In the transactivation assay, the ORFs of CsSPL3 and CsSPL14 were cloned into the pGBKT7 vector and transformed separately into yeast strain Y187 (Clontech). The empty vector pGBKT7 was used as the negative control. All the transformants were cultured on selective synthetic drop-out (SD) media of SD/–Trp, SD/–Trp/–His, and SD/–Trp/–Ade. For detection of the transactivation region in CsSPL14, a series of CsSPL14 deletions were inserted into pGBKT7 and transformed into yeast Y187 strain, and the transformants were cultured on the same selective media as noted above.

### Yeast two-hybrid screening and bimolecular fluorescence complementation (BiFC) assays

For the yeast two-hybrid (Y2H) assays, the full-length CsSPL3 and the fragment of CsSPL14 containing the SBP domain without the transactivation region were inserted into pGBKT7 to generate pBD-*SPL3*/*SPL14*. These were used as baits to screen the cDNA library to identify proteins that interacted with CsSPL3 and CsSPL14, respectively. The potential interacting proteins were amplified and inserted into the pGADT7 vector. They were then co-transformed with pBD-*SPL*s into AH109 (Clontech) and selected on SD/–Trp/–Leu. The interactions were evaluated on SD/–Trp/–Leu/–His/–Ade with X-α-galactosidase.

For BiFC analysis, PCR-amplified *CsSPL14* and the gene encoding the CsSPL14-interacting protein were inserted into pCL112 and pCL113 vectors, which carried the split N-terminal and C-terminal of YFP, respectively. *Agrobacterium tumefaciens* GV3101 carrying the experimental combinations of constructs were co-infiltrated into *Nicotiana benthamiana* leaves with a final OD_600_=0.8. The fluorescent signal was detected in the infiltrated sections of leaves 3 d after infiltration, using an Olympus FLUOVIEW FV1000 confocal microscope (Olympus, Japan).

### Statistical analysis

Data from at least two biological and three technical replicates were used for statistical analysis. Significance was determined by pairwise comparisons using *t*-tests, or multiple comparisons using the SAS software.

## Results

### Overexpression of csi-miR156a enhanced the SE capability of citrus callus

The full-length *CsMIR156A* transcript and the corresponding DNA sequence were both 787 bp, with no intron present (GeneBank No. MF069254). The transcription start site was located 525 bp upstream of the mature csi-miR156a. As shown in Supplementary Fig. S1, the precursors of csi-miR156a (MIR156a) were conserved among varieties, being defined as the sequence of ~200 nt comprising the 100 nt upstream and the 100 nt downstream of the mature miR156a. Given that SE capability in most of citrus genotypes gradually declines during long-term subculture, we first investigated the expression profiles of miR156 and its target *SPL*s in short- (<1 year) and long-term (nearly 10 years) preserved wild kumquat calluses, using qRT-PCR. The results showed that miR156 possessed significantly higher expression in short-term preserved calluses than that in the long-term ones, whereas five *CsSPL*s (*CsSPL*2/4/7/8/14) exhibited the opposite expression pattern (see Supplementary Fig. S2). Taken together with previous evidence also showing that csi-miR156a accumulated at higher levels in embryonic than in non-embryonic calluses (Wu *et al.*, 2015), the results suggested that miR156 may modulate SE capability.

**Fig. 1.**
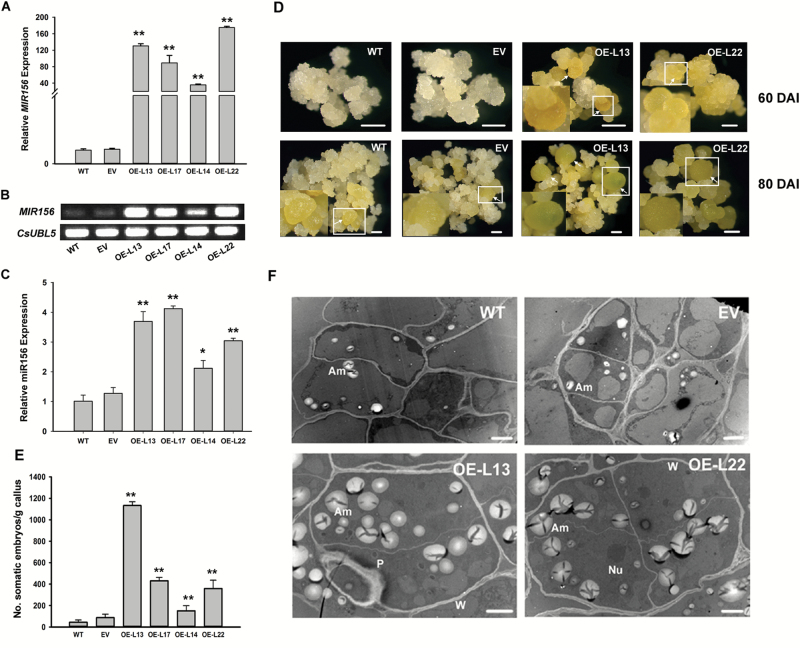
Overexpression of csi-miR156a enhances somatic embryogenesis (SE) capability in citrus *Fortunella hindsii*. (A, B) Relative expression of MIR156a (csi-miR156a precursor) in wild-type (WT), empty vector (EV), and overexpressed (OE) callus lines without SE induction, detected by qRT-PCR (A) and semi-qRT-PCR (B). *CsUBL5* was used as the endogenous control. (C) Relative expression of mature csi-miR156a in WT, EV, and OE lines detected by qRT-PCR. U6 was used as the internal control. (D) SE of WT, EV, and OE lines at 60 d and 80 d after induction (DAI). Calluses were induced on glycerol-containing medium, and sub-cultured at 20-d intervals. Somatic embryos formed at 60 DAI in the OE lines, whereas they did not form until 80 DAI in the WT and EV. Arrows indicate somatic embryos, and boxed regions are enlarged in the bottom corner of the figures to show the somatic embryos in close-up. Scale bars =1 mm. (E) Evaluation of SE capability in the WT, EV, and OE lines at 80 DAI. SE capability was measured as the number of somatic embryos formed per gram of fresh callus. Error bars represent standard deviation of three biological replicates. Statistically significant differences compared with the WT were determined by *t*-tests: *P<0.05; **P<0.01. (F) Cellular ultrastructure of the WT, EV, and OE lines. Am, amyloplast; Nu, nucleus; P, plastid; W, cell wall. Scale bars = 2 µm.

To validate the effect of csi-miR156a on citrus SE, *MIR156a* was overexpressed in calluses of wild kumquat driven by the *35S* promoter. Ten overexpressed (OE) callus lines and four control lines transformed with the empty vector (EV) were generated after screening by GUS staining and PCR amplification of the *Gus* and *HptⅡ* genes (see Supplementary Fig. S3). Four of the OE lines showed significantly higher expression levels of the precursor and the mature csi-miR156a than the WT and EV (Fig. 1A–C). At 60 DAI, globular embryos could be observed in the OE lines, whereas no obvious embryos formed in the control lines (Fig. 1D). The SE capability of the OE lines was significantly greater than the control lines, as compared by counting the number of embryos at 80 DAI (Fig. 1E). The ultrastructural observations showed that much bigger and more abundant amyloplasts accumulated in the embryonic cells of OE lines than in the control lines (Fig. 1F). Taken together, the results suggested that csi-miR156a enhanced the SE capability of citrus.

### Expression of *CsSPL3* and *CsSPL14* was highly correlated with citrus SE competence

**Fig. 2.**
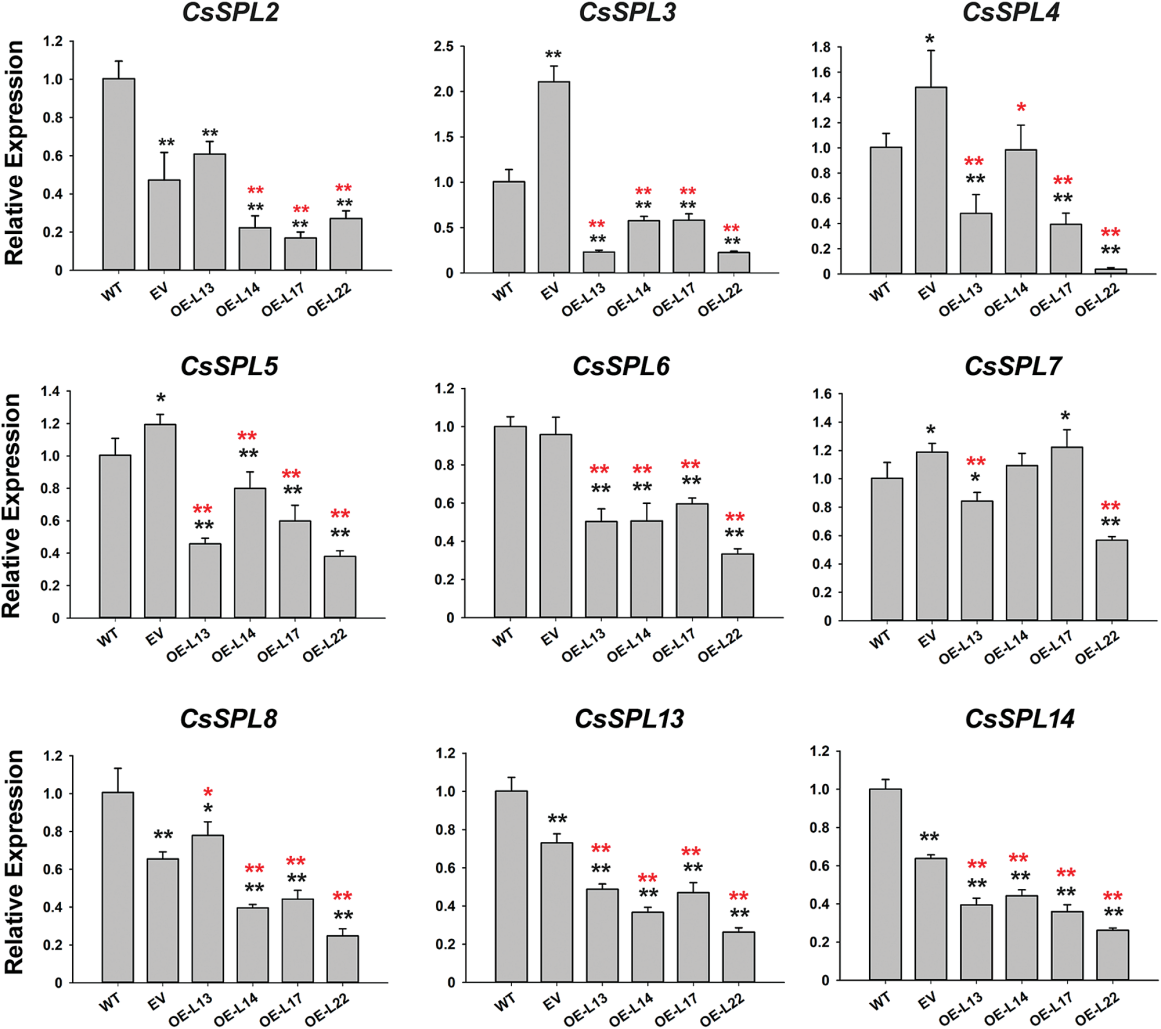
Transcript levels of csi-miR156a-targeted *CsSPL*s in citrus *Fortunella hindsii* detected by qRT-PCR. Relative expression of *CsSPL*s in wild-type (WT) and transgenic callus lines without SE induction. Nine *CsSPL*s were predicted as potential targets of csi-miR156a, of which five were down-regulated in csi-miR156a-overexpressed callus lines. EV, empty vector; OE-L13, OE-L14, OE-L17, and OE-L22 are the four selected overexpression lines. *CsUBL5* was used as the endogenous control. Error bars represent standard deviations of three biological replicates. Statistically significant differences were determined by *t*-tests: *P<0.05, **P<0.01. Black asterisks indicate comparisons with the WT, red asterisks indicate comparisons with the EV.

Out of the nine potential target *CsSPL* genes predicted by Liu *et al.* (2017), five were significantly down-regulated in all the miR156 OE callus lines relative to both the WT and EV, namely *CsSPL3* (*Cs1g07620*), *CsSPL5* (*Cs2g23550*), *CsSPL6* (*Cs5g12260*), *CsSPL13* (*orange 1.1t02265*), and *CsSPL14* (*orange 1.1t02597*); the other four showed inconsistently different expression profiles among the tested OE lines as compared with the WT and EV (Fig. 2). However, expression of most of the *CsSPL*s in the EV was either significantly up- or down-regulated relative to the WT, with only *CsSPL6* showing no change. In addition, the expression levels of *CsSPL3* and *CsSPL14* were highly correlated with the SE competence of the different citrus varieties. *CsSPL3* was significantly less abundant in calluses of the four SE-capable cultivars (AL, NH, V, and AS) than in the one with weak SE capability (WK) and the recalcitrant G1, except that the expression difference between V and G1 was insignificant. *CsSPL14* showed a similar expression pattern to *CsSPL3* except for relatively high abundance in AS, which resulted in an insignificant difference between G1 and AS (see Supplementary Fig. S4). These exceptions might be partially explained by the differing genetic backgrounds among these genotypes. On the basis of these results, we speculated that miR156-mediated regulation of SE induction in citrus callus was mainly through *CsSPL3* and *CsSPL14*.

A phylogenetic tree was constructed using the predicted miR156-targeted SPL protein sequences from Arabidopsis and rice. The phylogenetic relationship showed that CsSPL3 was clustered with OsSPL13 belonging to the SPL3 clade (represented by AtSPL3), whereas CsSPL14 was grouped with AtSPL13 (see Supplementary Fig. S5A). In the Arabidopsis transient protoplast transformation system, CsSPL3 and CsSPL14 fused with GFP both localized to the nucleus, and overlapped with the nucleus-localized marker, i.e. OsGhd7-CFP fusion proteins (Supplementary Fig. S5B), confirming their putative roles as transcription factors. In the yeast system, CsSPL3 and CsSPL14 were individually fused with the GAL4 DNA-binding domain in the vector pGBKT7. Yeast cells transformed with pGBKT7-CsSPL14 showed strong growth in the selective media that lacked adenine and histidine, whereas those transformed with pGBKT7-CsSPL3 and the empty vector failed to survive (Supplementary Fig. S5C), confirming the transactivation activity of CsSPL14 but not that of CsSPL3.

### Individual suppression of *CsSPL3* and *CsSPL14* promoted SE initiation from citrus callus

**Fig. 3.**
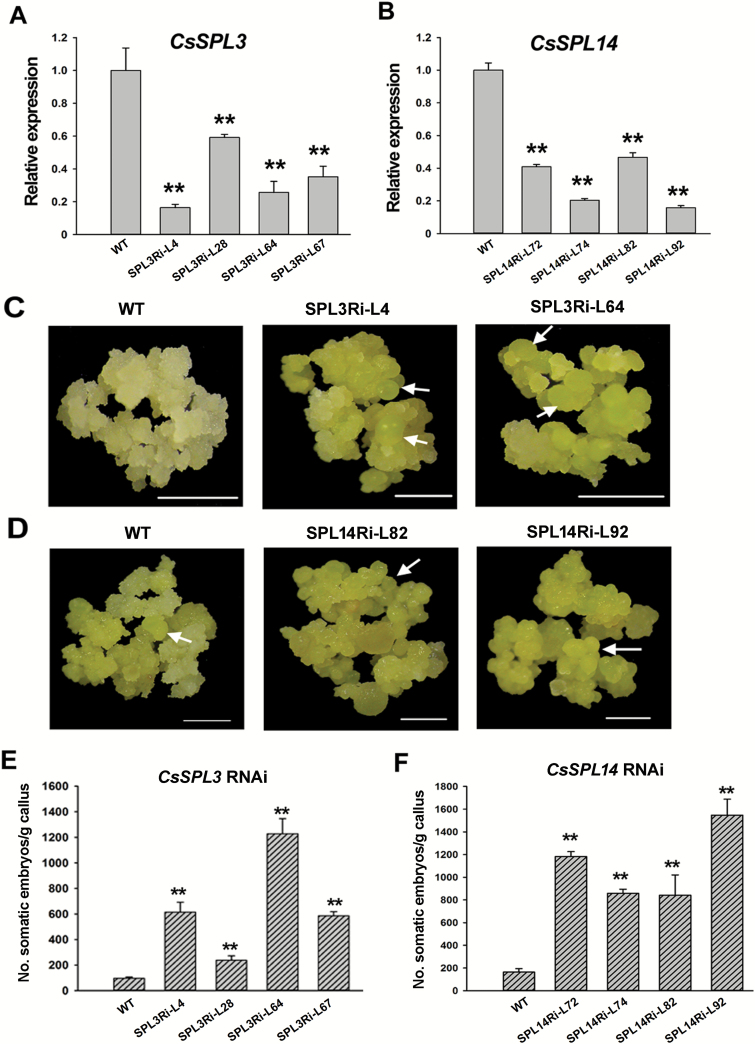
RNAi suppression of *CsSPL3* and *CsSPL14* enhances somatic embryogenesis (SE) capability in citrus *Fortunella hindsii*. (A, B) Transcript abundance of *CsSPL3* (A) and *CsSPL14* (B) in wild-type (WT) and transgenic callus lines. SPL3Ri-L4, SPL3Ri-L28, SPL3Ri-L64, and SPL3Ri-L67 are *CsSPL3* RNAi callus lines, while SPL14Ri-L72, SPL14Ri-L74, SPL14Ri-L82, and SPL14Ri-L92 are *CsSPL14* RNAi callus lines. *CsUBL5* was use as the endogenous reference gene. (C, D) SE of the WT compared with the *CsSPL3* (C) and the *CsSPL14* (D) RNAi lines at 80 d after induction (DAI), when the somatic embryos formed in the RNAi lines. Arrows indicate somatic embryos. Scale bars = 1mm. (E, F) Evaluation of SE capability in the WT and RNAi lines *CsSPL3* (E) and *CsSPL14* (F) at 100 DAI. Error bars represent standard deviation of three biological replicates. SE capability was measured as the number of somatic embryos formed per gram of fresh callus. Statistically significant differences compared with the WT were determined by *t*-tests (**P<0.01).

To test whether *CsSPL*s were involved in citrus SE, *CsSPL3* and *CsSPL14* were knocked-down individually by RNAi in wild kumquat calluses, resulting in independent transformed lines (see Supplementary Fig. S6). qRT-PCR analysis of the calluses without SE induction (cultured in MT medium) showed that *CsSPL3* expression decreased by 40–80% in all four lines, whilst *CsSPL14* decreased by 50–80% compared to the WT (Fig. 3 A, B). Interestingly, *CsSPL3* dramatically decreased in the *CsSPL14* RNAi lines, whereas *CsSPL14* had no significant expression change in the *CsSPL3* RNAi lines (Supplementary Fig. S7). Examination of the other seven *SPL*s in the *CsSPL3* and *CsSPL14* RNAi lines indicated that the accumulation of several *SPL*s had been changed (Supplementary Fig. S7). For example, *CsSPL6* was significantly down-regulated in both the *CsSPL3* RNAi lines, while *CsSPL2* and *CsSPL5* were up-regulated in SPL3Ri-28 and SPL14Ri-72, respectively. Only *CsSPL8* and *CsSPL13* showed insignificant differences between all the RNAi lines and WT (Supplementary Fig. S7).

At 80 DAI, somatic embryos were observed in both the *CsSPL3* and *CsSPL14* knock-down lines, whereas the controls rarely produce embryos (Fig. 3 C, D). At 100 DAI, the embryos in the RNAi lines were obviously bigger than the WT (see Supplementary Fig. S8). The number of embryos formed in the knock-down callus lines was significantly higher than the controls, proving that individual suppression of *CsSPL3* and *CsSPL14* also enhanced the SE capability of citrus callus (Fig. 3 E, F). These results showed that the expression levels of *CsSPL3* and *CsSPL14* were negatively correlative to SE initiation in citrus.

### Genome-wide identification of csi-miR156a-responsive genes during SE initiation

**Fig. 4.**
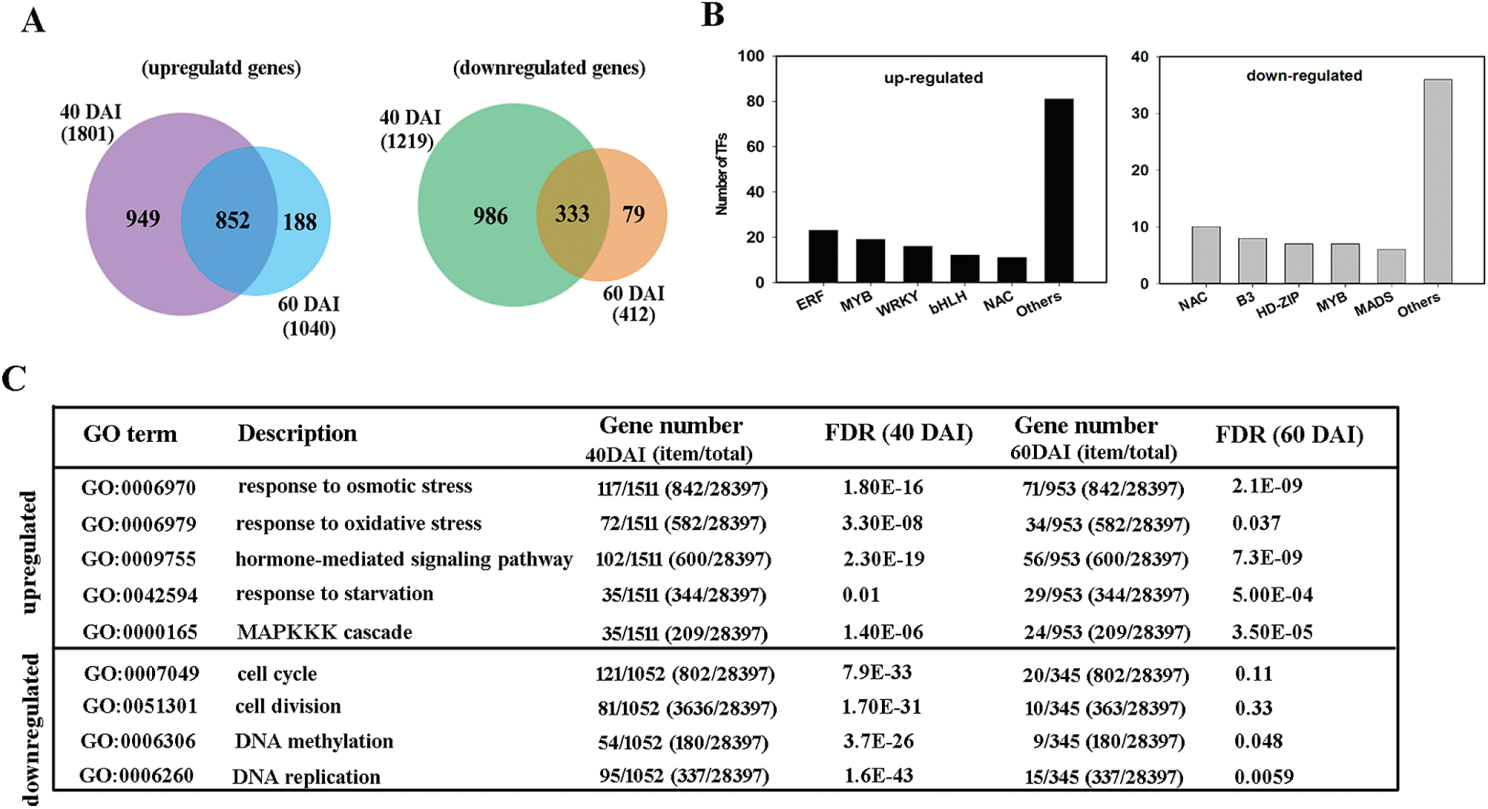
Identification of genes responsive to overexpression of csi-miR156a during somatic embryogenesis (SE) in citrus *Fortunella hindsii*. RNA-seq was conducted using calluses of the csi-miR156 overexpression line and the wild-type (WT) at 40 and 60 d after induction (DAI), the stages prior to and at the presence of somatic embryos, respectively. The WT was used as the control. (A) Overlapping differentially expressed genes (DEGs) between 40 and 60 DAI. The numbers in parentheses indicate the total DEGs at each stage. (B) Number of transcription factors (TFs) identified among up- and down-regulated genes in the RNA-seq data. (C) Selected significantly enriched GO terms for biological process. The up- and down-regulated genes were analysed for over-represented GO terms at the two stages. ‘item/total’, the number of genes enriched in the selected GO term/the total number of genes in all GO terms. Numbers in parentheses indicate the background counterparts. FDR, false discovery rate.

To explore the downstream pathway of miR156-*SPL*-mediated regulation of SE capability, RNA-seq comparisons were conducted between two of the miR156-OE lines and the WT lines at 40 and 60 DAI, immediately before and after substantial embryo formation in the OE lines. After filtering, nearly 90% of the clean reads in each sample were aligned to the reference clementine genome (see Supplementary Table S2). The correlation coefficients between replicate pairs were never less than 0.936, with a maximum of 0.979 (Supplementary Table S3). A total of 3021 (1801 up, 1219 down) and 1452 (1040 up, 412 down) DEGs were identified at 40 and 60 DAI, respectively, in the OE lines as compared with the control. For the two stages, the majority of DEGs overlapped (1185, 852 up, 333 down) (Fig. 4A). Moreover, 224 DEGs were annotated as transcription factors, and among the up-regulated genes the five most frequently represented families were ERF, WRKY, bHLH, NAC, and bZIP, while among the down-regulated genes they were NAC, B3, MYB, HD-ZIP, and MADS (Fig. 4B). According to the GO enrichment analysis, 435 and 338 biological processes were significantly enriched among genes up-regulated in the OE lines at 40 and 60 DAI, respectively (Supplementary Table S4). In particular, response to stress was found to be over-represented at both stages, especially responses to osmotic and oxidative stress (Fig. 4C); also over-represented were the response to starvation, the MAPKKK cascade, and the signaling pathways related to hormones, including jasmonic acid, ethylene, and gibberellic acid (Fig. 4C, Supplementary Table S4). Among the down-regulated genes in the OE lines, 348 and 48 biological processes were enriched at 40 and 60 DAI, respectively (Supplementary Table S4). Epigenetic-related processes, i.e. DNA methylation and DNA replication, were enriched at both stages; cell cycle processes, especially cell division, were over-represented at 40 but not 60 DAI (Fig. 4C).

**Fig. 5.**
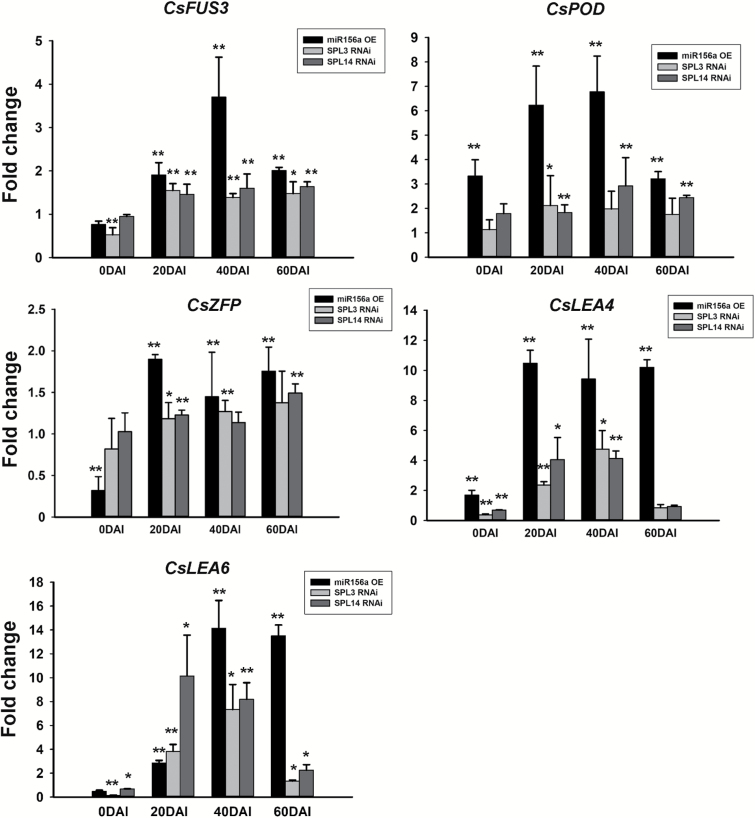
Fold-change in expression levels of SE-related genes in the transgenic lines of citrus *Fortunella hindsii* compared with the wild-type (WT). CsFUS3, FUSCA3 transcription factor; CsPOD, peroxidase; CsZFP, zinc-finger CCCH domain-containing protein; CsLEA, late-embryogenesis abundant protein. Error bars indicated standard deviation of two biological replicates (represented by two transgenic lines), each containing at least three technical replicates. *CsUBL5* was used as the internal reference. Statistically significant differences compared with the WT were determined by *t*-tests: *P<0.05, **P<0.01.

Specifically, some well-known SE-related genes, such as *LEA* (*late embryogenesis abundant*) and *FUS3* (*FUSCA3*, a B3 domain transcription factor gene) with higher accumulation during the citrus SE process (Ge *et al.*, 2012), were up-regulated in the miR156-OE callus lines at both stages. Similarly, a zinc-finger CCCH domain-containing protein gene (*ZFP*) and a peroxidase gene (*POD*) were also significantly up-regulated in the miR156-OE lines, indicating that both the genes were involved in the regulatory pathway mediated by the miR156-*SPL* modules. We further investigated the expression of *LEA*, *FUS3*, *ZFP*, and *POD* in the miR156a OE and *CsSPL* RNAi lines during SE initiation by qRT-PCR (Fig. 5). Cs*FUS3* and *CsLEA6* were significantly up-regulated throughout the SE induction process (from 20 to 60 DAI) in both the miR156-OE and individual *CsSPL3* and *CsSPL14* RNAi lines. Increased transcript abundance of *CsPOD* was detected throughout the SE induction process (from 20 to 60 DAI) in both the miR156a OE and *CsSPL*14 RNAi lines, but only at 20 DAI in the *CsSPL*3 RNAi lines. *CsZFP* showed higher accumulation in all transgenic lines at 20 DAI and in miR156-OE lines at both 40 and 60 DAI compared with the WT, as well as in the *CsSPL*3 RNAi lines at 40 DAI and in the *CsSPL14* RNAi lines at 60 DAI. *CsLEA4* was consistently up-regulated in all transgenic lines at 20 and 40 DAI, but no significant changes were detected in RNAi lines at 60 DAI.

### Identification of CsSPL-interacting proteins

**Fig. 6.**
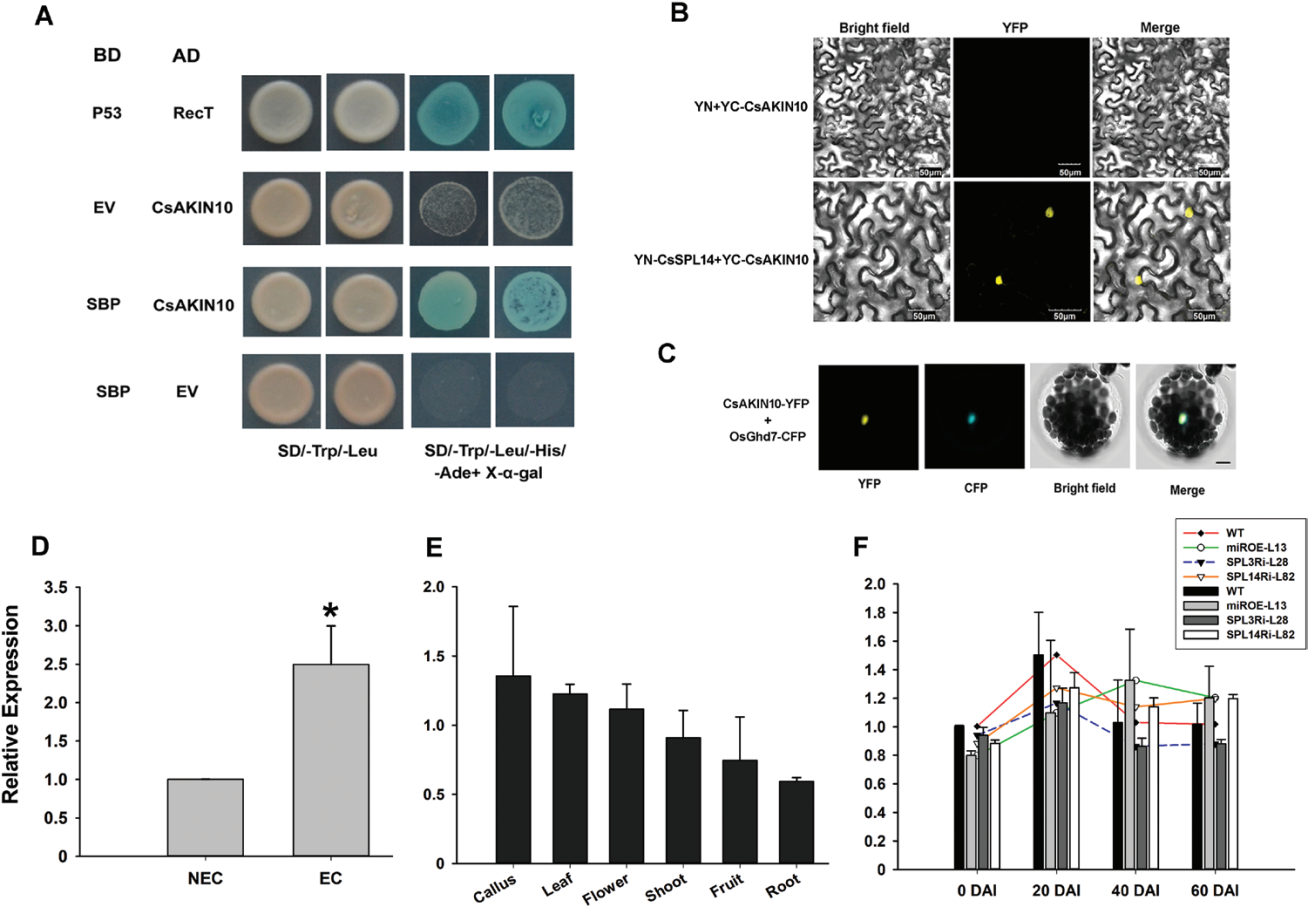
Identification of the CsSPL14-interacting protein. (A) The interaction between CsAKIN10 and CsSPL14 verified by yeast two-hybrid assays. Clones grown on synthetic drop-out selection medium that lacked Trp and Leu (SD/–Trp/–Leu) were detected by synthetic drop-out selection medium that lacked Trp, Leu, His, and Ade (SD/–Trp/–Leu/–His/–Ade) with X-α-gal. Co-transformation of BD-p53 and AD-RecT was used as the positive control. EV, empty vector. (B) CsAKIN10 was confirmed to interact with full-length CsSPL14 by BiFC assays. The construct of CsSPL14 fused with the N-terminal of YFP was co-infiltrated with CsAKIN10 fused with the C-terminal of YFP into tobacco leaves. The co-infiltrated leaves were photographed after 3 d. (C) Sub-cellular localization analysis of CsAKIN10. The CsAKIN10-YFP construct was co-transformed with OsGhd7-CFP, a positive control for nuclear localization, into Arabidopsis mesophyll protoplasts. The images were captured under yellow (YFP) and cyan fluorescence (CFP), and bright light, and the merged image is also shown. Scale bar = 7.5 µm. (D) Expression levels of CsAKIN10 in non-embryonic callus (NEC) and embryonic callus (EC) in ‘Valencia’ sweet orange, which has high SE competence. Statistical significance was determined by a *t*-test, * *P*<0.05. (E) Expression levels of CsAKIN10 in different tissues of ‘Valencia’. (F) Expression levels of CsAKIN10 in miR156 overexpression and CsSPL3 and CsSPL14 RNAi callus lines during SE induction in *Fortunella hindsii*. miROE-L13, miR156 overexpression line; SPL3Ri-L28, CsSPL3 RNAi line; SPL14Ri-L82, CsSPL14 RNAi line. DAI, days after SE induction.

Y2H screening assays were conducted to identify the SPL-interacting proteins that we believed to be involved in SE. However, no positive clone was obtained with CsSPL3 as the bait, whilst the full-length CsSPL14 protein exhibited self-activation activity and toxicity to yeast growth (see Supplementary Fig. S5), leaving only the SBP domain (88–162 amino acids) as a usable bait for screening the interacting proteins from the citrus cDNA library of ECs and somatic embryos (Supplementary Fig. S9). Sequencing of the positive clones from Y2H screening determined that CsAKIN10 (SNF1-related protein kinase catalytic subunit alpha KIN10, Cs6g21650.3) interacted with the SBP domain of CsSPL14 (Fig. 6A). BiFC assays confirmed the interaction between full-length CsSPL14 and CsAKIN10 in tobacco leaves, in which the YFP signal was reconstituted in co-transformation of YN-CsSPL14 (YFP N-terminus) and YC-CsAKIN10 (YFP C-terminus). By contrast, co-transformation of YN (empty vector) and YC-CsAKIN10 failed to generate a YFP signal (Fig. 6B). Subcellular localization analysis showed that CsAKIN10 was also localized to the nucleus (Fig. 6C).

Expression of *CsAKIN10* was detected in different tissues of the genotype ‘Valencia’ sweet orange with high SE competence (Fig. 6D, E). The results showed that the transcript level of *CsAKIN10* in ECs was significantly higher than in non-embryonic calluses (NECs) (Fig. 6D). However, accumulation of *CsAKIN10* showed no significant differences among different tissues, namely ECs, flowers, fruit, leaves, roots, and shoots (Fig. 6E). We further examined the expression patterns of *CsAKIN10* in the WT and the transgenic calluses (including csi-miR156 OE, *CsSPL3*, and *CsSPL14* RNAi lines). No significant differences were found between the WT and transgenic lines, and their expression patterns were similar, with an increase from 0 to 20 DAI, then a decrease at 40 DAI, and stable expression at 60 DAI (Fig. 6F).

## Discussion

As a conserved and well-studied miRNA family in plants, miR156s are known to regulate diverse aspects of plant development (Wang and Wang, 2015), and in particular miR156-mediated regulation of phase transition is highly conserved within the plant kingdom (Chuck *et al.*, 2007; Wang *et al.*, 2009, 2011; Sun *et al.*, 2013). Among the miR156 family members identified in citrus, csi-miR156a is the most abundantly accumulated miRNA both in the embryogenic callus and during SE (Wu *et al.*, 2015). In the present study, overexpression of csi-miR156a was found to enhance the SE capability of citrus callus, confirming the promotive effect of miR156 on SE. RNA-seq analysis suggested that overexpression of miR156 might trigger biological processes such as stress responses and the hormone signaling pathway to promote SE. Among the ten *CsSPL*s targeted by miR156 (Liu *et al.*, 2017), *CsSPL3* and *CsSPL14* were identified to be the potential downstream target genes that regulated SE. Individual knock-down of the two *SPL* targets also enhanced the SE capability, proving the negative effect of *SPL* targets on SE. CsSPL3 and CsSPL14 were characterized, and CsAKIN10 was identified to be interactive with CsSPL14. Based on the findings of this study, the possible function of the miR156-*SPL* modules in SE can be discussed.

### The novel function of miR156-*SPL* modules in SE

In Arabidopsis, numerous miRNAs regulating SE induction have been identified, including miR156 (Szyrajew *et al.*, 2017). However, previous studies have been focused at the transcription level and on the functional annotation of the miRNAs related to SE, and have lacked experimental investigation of the regulatory roles of miRNAs in SE. In Arabidopsis, miR156-mediated repression of *SPL* targets was recognized as an intrinsic time-cue that regulates the shoot regenerative (organogenesis) capacity of leaf-derived calluses (Zhang *et al.*, 2015, 2017). Recently, *GhmiR157*/*GhSPL10* was reported to modulate cell de-differentiation and callus proliferation in cotton explants, and thus to affect SE efficiency (Wang *et al.*, 2018). In citrus, expression levels of miR156 are much higher in ECs than NECs (Wu *et al.*, 2015), in agreement with our findings of significantly higher accumulation of miR156 in short-term sub-cultured calluses (with strong SE capacity) than in long-term sub-cultured calluses (with weak SE capacity) (see Supplementary Fig. S2). Accordingly, in this study, miR156 was confirmed to promote SE of citrus through repression of target *CsSPL* genes.

Unlike Arabidopsis, cotton, and the vast majority of other plant species where exogenous cytokinin or auxin is required for regeneration, a glycerol medium without hormone supplement is sufficient for induction of citrus SE (Wu *et al.*, 2009b). For *in vitro* shoot regeneration of Arabidopsis, *SPL9* group genes (including *AtSPL2*, *9*, *10*, *11*, *13*, and *15*), rather than the *SPL3* group (including *AtSPL3*, *4* and *5*) have been identified to be the functional targets downstream of miR156 (Zhang *et al.*, 2015). In addition, *AtSPL10* and *AtSPL11* were previously characterized to regulate Arabidopsis zygotic embryogenesis (Nodine and Bartel, 2010). In our study, however, two non-SPL9 clustered *SPL*s (*CsSPL3* and *14*), were demonstrated to be involved in the regulation of citrus SE, indicating that different *SPL*s might contribute to distinct types of regeneration. Citrus csi-miR156a is shorter (20 nt) than cotton GhmiR157a (21 nt), and contains different nucleotides at three positions. Cotton GhSPL10 is clustered with AtSPL4/5 but not AtSPL3 (Cai *et al.*, 2018), whereas CsSPL3 was clustered with AtSPL3/4/5 (see Supplementary Fig. S5A). Our finding that miR156 promoted SE of citrus callus, i.e. the differentiation of *in vitro* cultured cells, is in agreement with the reported finding that miR157-targed GhSPL10 promotes de-differentiation and callus proliferation (Wang *et al.*, 2018).

OsSPL13, which was clustered with CsSPL3 (Supplementary Fig. S5A), has been reported to modulate grain size, shape, and yield, and to be involved in tiller and panicle branching in rice (Si *et al.*, 2016). In Arabidopsis, *AtSPL3* has been reported to shorten the juvenile period, and to promote early flowering and the transition of floral meristem identity (Wu and Poethig, 2006; Yamaguchi *et al.*, 2009; Xu *et al.*, 2016). Overexpression of *CiSPL5*, the orthologous counterpart in the *Citrus clementina* genome with CsSPL3, was found to promote flowering in Arabidopsis (Shalom *et al.*, 2015), suggesting a similar function of CsSPL3 to AtSPL3 in plants. However, the involvement of *CsSPL3* in SE as demonstrated in our study might suggest a novel function of SPL3-group genes in regulating SE of *in vitro* cultured calluses.

AtSPL13 acts as the major member in SPL9 group that regulates shoot phase transition and development, and is also one of the SPLs that promotes expression of *MIR172* genes (Xu *et al.*, 2016). Repression of AtSPL13 by miR156 is essential for the normal post-germinative switch from the cotyledon stage to the vegetative-leaf stage in Arabidopsis (Martin *et al.*, 2010*a*, 2010*b*). CsSPL14, which is orthologous with AtSPL13 in the SPL9 group, was also found to be a repressor of SE as was the SPL3 group gene *CsSPL3*. Interestingly, the abundance of *CsSPL3* was reduced by suppression of *CsSPL14*, but down-regulation of *CsSPL3* did not alter *CsSPL14* expression (see Supplementary Fig. S7), suggesting that *CsSPL14* probably regulates expression of *CsSPL3* during SE.

### Genes responding to miR156 overexpression and the triggering of SE

Transcriptomic profiles of calluses were compared between the miR156-overexpressing and the WT lines at 40 and 60 DAI, i.e. the stages before and at the presence of somatic embryos, in order to try to deduce the reasons for higher SE capability. Interestingly, the number of DEGs at 40 DAI was nearly twice that at 60 DAI (Fig. 4A), indicating more dramatic changes in gene expression during SE induction than during post-embryo formation. Previous studies have established that phytohormone-related pathways and stress responses are triggered during SE in different plant species (Jin *et al.*, 2014; Zheng *et al.*, 2016). Furthermore, among the genes targeted by miRNAs differentially expressed during SE in Arabidopsis, the majority are also related to phytohormones and stress responses (Szyrajew *et al.*, 2017). Likewise, in our study of responses to miR156 overexpression in citrus we found that the enriched biological processes of the genes included mainly hormone-mediated signaling pathways and stress responses, and in particular responses to starvation, and osmotic and oxidative stresses were over-represented among the up-regulated genes (Fig. 4C). Glycerol in the citrus SE-induction medium is recognized as an ineffective carbohydrate source and also as an osmotic substance, whilst SE as a differentiation process may require the involvement of endogenous phytohormones, which would explain the enrichment of hormone-mediated signaling pathways and stress responses.

Consistent with a previous finding that extensive TFs are associated with SE in Arabidopsis (Gliwicka *et al.*, 2013), 224 differentially expressed TF genes were identified in our study. The most abundant TFs related to competency of SE (e.g. *bHLH*, *NAC*, *WRKY*, *MYB*, *MADS*) in Arabidopsis were highly homologous to those found in our study (Fig. 4B), indicating that these TFs might also regulate SE in citrus. Several well-known SE-associated genes, including *CsFUS3*, *CsLEA*, *CsZPF*, and *CsPOD*, showed similar expression patterns of up-regulation in both the miR156-OE and *CsSPL3*/*14* RNAi lines after SE induction, indicating that many important SE genes began to be activated after SE induction, and maintained high-levels of expression through to the emergence of globular embryos (60 DAI). Thus, we assume that miR156-*CsSPL* modules might act upstream of these SE-related genes. Although the globular embryos could not develop and form whole plants, these important SE genes were expressed in the phase of induction, suggesting that they are required for SE initiation, at least for globular embryo formation. The detailed interactive relationship that exists between miR156-*CsSPL*s and the SE-related TF genes would be a topic of great interest and importance for further research to explore the mechanisms by which miR156-*CsSPL* modules regulate SE.

In general, *FUS3 and LEA* are predominantly expressed at the late stages of zygotic embryogenesis (Wise and Tunnacliffe, 2004; Braybrook and Harada, 2008). However, in the SE system of Arabidopsis, expression of *FUS3* and *LEA* are also observed in embryonic cultures and somatic embryos (Ikeda-Iwai *et al.*, 2002). Similarly, these two genes were found to be constantly accumulated during the SE induction phase in ‘Valencia’ sweet orange (Ge *et al.*, 2012), which is in agreement with our findings. In addition, we found that other important SE genes such as *LEC1*, *ABSCISIC ACID INSENSITIVE 3* (*ABI3*), and *ABI5* were significantly up-regulated according to the RNA-seq data, in agreement with the findings of Ge *et al.* (2012). However, *WOX1* and *WOX2* were significantly down-regulated in the miR156 overexpression lines, which is inconsistent with previous reports that *WOX*s are positive regulators of SE, especially for early embryo induction (Palovaara *et al.*, 2010; Gambino *et al.*, 2011; Rupps *et al.*, 2016). Other key SE genes such as *AGL15*, *SERK* and *BBM* reported in other SE systems (Zheng *et al.*, 2013; Rupps *et al.*, 2016) were not significantly differentially expressed between the miR156-OE lines and the WT. These results suggest that SE induction in different species might be differentially regulated by distinct genes.

### Possible role of the CsSPL14-interacting CsAKIN10 protein in citrus SE

In our study, the CsAKIN10 protein was identified to be a co-localized and interactive with CsSPL14 in the nucleus. In Arabidopsis, *AKIN10* encodes a catalytic α-subunit of Snf1-related kinase1 (SnRK1), which is also localized in the nucleus (Bitrián *et al.*, 2011). Through interaction and phosphorylation, AKIN10 stabilizes the B3-domain transcription factor FUS3 to regulate both the embryonic-to-vegetative and vegetative-to-reproductive phase transitions (Tsai and Gazzarrini, 2012). Interestingly, *CsFUS3* was significantly up-regulated in both the miR156-OE and individual *CsSPL3/14* RNAi lines after SE induction (Fig. 5). As FUS3 is known to be essential for both late-zygotic embryogenesis and SE in Arabidopsis (Gaj *et al.*, 2005; Braybrook and Harada, 2008; Suzuki and McCarty, 2008), we propose that the CsAKIN10–FUS3 interaction in the calluses might increased CsFUS3 expression to promote SE induction. However, no interaction was detected between CsAKIN10 and CsFUS3 according to BiFC assays (see Supplementary Fig. S10). The pathway downstream of miR156a–CsSPL–CsAKIN10 remains to be identified in the future.

As well as the pathway downstream of the miR156-CsSPL module, it is also of interest to identify the upstream factor that controls the effect of miR156 in SE. It has been reported that two AGAMOUS-like proteins, AGL15 and AGL18, might form a complex and bind to the promoters of *MIR156*, thus inducing *MIR156* to act as a floral repressor in Arabidopsis (Serivichyaswat *et al.*, 2015). In addition, *AGL15* and *AGL18* have been confirmed to promote SE in soybean and Arabidopsis (Thakare *et al.*, 2008; Zheng *et al.*, 2013; Zheng and Perry, 2014). In our study, the RNA-seq analysis indicated that neither *AGL15* nor *AGL18* had any significant changes in expression in the miR156-OE lines compared to the WT, suggesting at least that *AGL*s do not act downstream of the miR156-CsSPL module. The possibility that AGL or other proteins act upstream of miR156 to modulate SE remains to be explored.

In summary, in this study a novel regulatory role for miR156 in SE was confirmed in the citrus *in vitro* cultured callus system. Identification of the miR156–CsSPL3 and miR156–CsSPL14–CsAKIN10 cascades provides a preliminary regulatory pathway for SE that might ultimately activate hormone-signalling pathways and stress responses during SE initiation. Our results provide promising candidate genes for the maintenance and enhancement of SE capability; however, the downstream regulatory factors remain to be elucidated before the complete pathway can be determined and we can fully understand the miR156-mediated modulation of cell pluripotency.

## Supplementary data

Supplementary data are available at *JXB* online.

Fig. S1. DNA sequence alignment of the csi-miR156a gene *CsMIR156A* in different citrus genotypes with various SE capacities.

Fig. S2. Expression levels of csi-miR156 and *CsSPL*s in long- and short-term preserved calluses.

Fig. S3. Generation and characterization of *MIR156a* precursor transgenic callus lines.

Fig. S4. Expression patterns of *CsSPL3* and *CsSPL14* in calluses of different citrus genotypes.

Fig. S5. Phylogenetic tree, subcellular localization, and transactivation activities of CsSPL3 and CsSPL14.

Fig. S6. Generation of *CsSPL3* and *CsSPL14* RNAi callus lines.

Fig. S7. Detection of transcript abundance of predicted miR156-targeted *CsSPL*s in the *CsSPL3* and *CsSPL14* RNAi lines.

Fig. S8. Somatic embryos formed in the RNAi lines at 100 DAI.

Fig. S9. Yeast growth and self-activation tests of truncated CsSPL14.

Fig. S10. Lack of interaction between CsAKIN10 and CsFUS3 as determined by BiFC assays.

Table S1. Primers used in this study.

Table S2. Summary of RNA-seq data.

Table S3. Correlation coefficients between biological replicates for the RNA-seq data.

Table S4. Enriched GO terms (biological process) for differentially expressed genes between the miR156-overexpression callus lines and the wild-type.

Supplementary Tables and Figuers

Supplementary Table S4

## References

[CIT0001] Anders S, Pyl PT, Huber W 2015 HTSeq—a Python framework to work with high-throughput sequencing data. Bioinformatics 31, 166–169. 25260700 10.1093/bioinformatics/btu638 PMC4287950

[CIT0002] Bitrián M, Roodbarkelari F, Horváth M, Koncz C 2011 BAC-recombineering for studying plant gene regulation: developmental control and cellular localization of SnRK1 kinase subunits. The Plant Journal 65, 829–842. 21235649 10.1111/j.1365-313X.2010.04462.x

[CIT0003] Braybrook SA, Harada JJ 2008 LECs go crazy in embryo development. Trends in Plant Science 13, 624–630. 19010711 10.1016/j.tplants.2008.09.008

[CIT0004] Cai C, Guo W, Zhang B 2018 Genome-wide identification and characterization of SPL transcription factor family and their evolution and expression profiling analysis in cotton. Scientific Reports 8, 762. 29335584 10.1038/s41598-017-18673-4 PMC5768680

[CIT0005] Cao H, Zhang J, Xu J, Ye J, Yun Z, Xu Q, Xu J, Deng X 2012 Comprehending crystalline β-carotene accumulation by comparing engineered cell models and the natural carotenoid-rich system of citrus. Journal of Experimental Botany 63, 4403–4417. 22611233 10.1093/jxb/ers115 PMC3421982

[CIT0006] Chávez-Hernández EC, Alejandri-Ramírez ND, Juárez-González VT, Dinkova TD 2015 Maize miRNA and target regulation in response to hormone depletion and light exposure during somatic embryogenesis. Frontiers in Plant Science 6, 555. 26257760 10.3389/fpls.2015.00555 PMC4510349

[CIT0007] Chen CJ, liu Q, Zhang YC, Qu LH, Chen YQ, Gautheret D 2011 Genome-wide discovery and analysis of microRNAs and other small RNAs from rice embryogenic callus. RNA Biology 8, 538–547. 21525786 10.4161/rna.8.3.15199

[CIT0008] Cheng YJ, Guo WW, Yi HL, Pang XM, Deng XX 2003 An efficient protocol for genomic DNA extraction from *Citrus* species. Plant Molecular Biology Reports 21, 177.

[CIT0009] Chuck G, Cigan AM, Saeteurn K, Hake S 2007 The heterochronic maize mutant *Corngrass1* results from overexpression of a tandem microRNA. Nature Genetics 39, 544–549. 17369828 10.1038/ng2001

[CIT0010] Cui LG, Shan JX, Shi M, Gao JP, Lin HX 2014 The *miR156*-*SPL9*-*DFR* pathway coordinates the relationship between development and abiotic stress tolerance in plants. The Plant Journal 80, 1108–1117. 25345491 10.1111/tpj.12712

[CIT0011] Du Z, Zhou X, Ling Y, Zhang Z, Su Z 2010 agriGO: a GO analysis toolkit for the agricultural community. Nucleic Acids Research 38, W64–W70. 20435677 10.1093/nar/gkq310 PMC2896167

[CIT0012] Duan YX, Guo WW, Meng HJ, Tao NG, Li DL, Deng XX 2007 High efficient transgenic plant regeneration from embryogenic calluses of *Citrus sinensis*. Biologia Plantarum 51, 212–216.

[CIT0013] Fang Y, Xie K, Xiong L 2014 Conserved miR164-targeted NAC genes negatively regulate drought resistance in rice. Journal of Experimental Botany 65, 2119–2135. 24604734 10.1093/jxb/eru072 PMC3991743

[CIT0014] Fehér A 2015 Somatic embryogenesis — stress-induced remodeling of plant cell fate. Biochimica et Biophysica Acta 1849, 385–402. 25038583 10.1016/j.bbagrm.2014.07.005

[CIT0015] Gaj MD, Zhang S, Harada JJ, Lemaux PG 2005 Leafy cotyledon genes are essential for induction of somatic embryogenesis of *Arabidopsis*. Planta 222, 977–988. 16034595 10.1007/s00425-005-0041-y

[CIT0016] Gambino G, Minuto M, Boccacci P, Perrone I, Vallania R, Gribaudo I 2011 Characterization of expression dynamics of WOX homeodomain transcription factors during somatic embryogenesis in *Vitis vinifera*. Journal of Experimental Botany 62, 1089–1101. 21127025 10.1093/jxb/erq349

[CIT0017] Ge XX, Chai LJ, Liu Z, Wu XM, Deng XX, Guo WW 2012 Transcriptional profiling of genes involved in embryogenic, non-embryogenic calluses and somatic embryogenesis of Valencia sweet orange by SSH-based microarray. Planta 236, 1107–1124. 22622359 10.1007/s00425-012-1661-7

[CIT0018] Germana MA, Lambardi M **eds** 2016 In vitro embryogenesis in higher plants. Methods in Molecular Biology Series, Springer Protocols, 1359. New York: Humana Press.

[CIT0019] Gliwicka M, Nowak K, Balazadeh S, Mueller-Roeber B, Gaj MD 2013 Extensive modulation of the transcription factor transcriptome during somatic embryogenesis in *Arabidopsis thaliana*. PLoS ONE 8, e69261. 23874927 10.1371/journal.pone.0069261 PMC3714258

[CIT0020] Gou JY, Felippes FF, Liu CJ, Weigel D, Wang JW 2011 Negative regulation of anthocyanin biosynthesis in *Arabidopsis* by a miR156-targeted SPL transcription factor. The Plant Cell 23, 1512–1522. 21487097 10.1105/tpc.111.084525 PMC3101539

[CIT0021] Guan Y, Li SG, Fan XF, Su ZH 2016 Application of somatic embryogenesis in woody plants. Frontiers in Plant Science 7, 938. 27446166 10.3389/fpls.2016.00938 PMC4919339

[CIT0022] Guo WW, Cai XD, Cheng YJ, Grosser J, Deng XX 2007a Protoplast technology and citrus improvement. In: Xu Z, Li J, Xue Y, Yang W, eds. Biotechnology and sustainable agriculture 2006 and beyond. Netherlands: Springer, 461–464.

[CIT0023] Guo WW, Li DL, Duan YX 2007b Citrus transgenics: current status and prospects. Transgenic Plant Journal 1, 202–209.

[CIT0024] Huang W, Peng S, Xian Z, Lin D, Hu G, Yang L, Ren M, Li Z 2017 Overexpression of a tomato miR171 target gene *SlGRAS24* impacts multiple agronomical traits via regulating gibberellin and auxin homeostasis. Plant Biotechnology Journal 15, 472–488. 27712008 10.1111/pbi.12646 PMC5362688

[CIT0025] Ikeda-Iwai M, Satoh S, Kamada H 2002 Establishment of a reproducible tissue culture system for the induction of Arabidopsis somatic embryos. Journal of Experimental Botany 53, 1575–1580. 12096096 10.1093/jxb/erf006

[CIT0026] Ikeuchi M, Ogawa Y, Iwase A, Sugimoto K 2016 Plant regeneration: cellular origins and molecular mechanisms. Development 143, 1442–1451. 27143753 10.1242/dev.134668

[CIT0027] Jiménez VM 2005 Involvement of plant hormones and plant growth regulators on *in vitro* somatic embryogenesis. Plant Growth Regulation 47, 91–110.

[CIT0028] Jin F, Hu L, Yuan D, Xu J, Gao W, He L, Yang X, Zhang X 2014 Comparative transcriptome analysis between somatic embryos (SEs) and zygotic embryos in cotton: evidence for stress response functions in SE development. Plant Biotechnology Journal 12, 161–173. 24112122 10.1111/pbi.12123

[CIT0029] Kou SJ, Wu XM, Liu Z, Liu YL, Xu Q, Guo WW 2012 Selection and validation of suitable reference genes for miRNA expression normalization by quantitative RT-PCR in citrus somatic embryogenic and adult tissues. Plant Cell Reports 31, 2151–2163. 22865195 10.1007/s00299-012-1325-x

[CIT0030] Li C, Zhang B 2016 MicroRNAs in control of plant development. Journal of Cellular Physiology 231, 303–313. 26248304 10.1002/jcp.25125

[CIT0031] Li J, Hou H, Li X, Xiang J, Yin X, Gao H, Zheng Y, Bassett CL, Wang X 2013 Genome-wide identification and analysis of the SBP-box family genes in apple (*Malus × domestica* Borkh.). Plant Physiology and Biochemistry 70, 100–114. 23771035 10.1016/j.plaphy.2013.05.021

[CIT0032] Lin Y, Lai Z 2013 Comparative analysis reveals dynamic changes in miRNAs and their targets and expression during somatic embryogenesis in longan (*Dimocarpus longan* Lour.). PLoS ONE 8, e60337. 23593197 10.1371/journal.pone.0060337 PMC3623967

[CIT0033] Liu J, Sheng L, Xu Y, Li J, Yang Z, Huang H, Xu L 2014 *WOX11* and *12* are involved in the first-step cell fate transition during de novo root organogenesis in *Arabidopsis*. The Plant Cell 26, 1081–1093. 24642937 10.1105/tpc.114.122887 PMC4001370

[CIT0034] Liu MY, Wu XM, Long JM, Guo WW 2017 Genomic characterization of miR156 and SQUAMOSA promoter binding protein-like genes in sweet orange (*Citrus sinensis*). Plant Cell, Tissue and Organ Culture 130, 103–116.

[CIT0035] Liu YZ, Liu Q, Tao NG, Deng XX 2006 Efficient isolation of RNA from fruit peel and pulp of ripening navel orange (*Citrus sinensis* Osbeck). Journal of Huazhong Agricultural University 25, 300–304 (in Chinese with English abstract).

[CIT0036] Liu Z, Ge XX, Wu XM, Kou SJ, Chai LJ, Guo WW 2013 Selection and validation of suitable reference genes for mRNA qRT-PCR analysis using somatic embryogenic cultures, floral and vegetative tissues in citrus. Plant Cell, Tissue and Organ Culture 113, 469–481.

[CIT0037] Luo YC, Zhou H, Li Y, Chen JY, Yang JH, Chen YQ, Qu LH 2006 Rice embryogenic calli express a unique set of microRNAs, suggesting regulatory roles of microRNAs in plant post-embryogenic development. FEBS Letters 580, 5111–5116. 16959252 10.1016/j.febslet.2006.08.046

[CIT0038] Martin RC, Asahina M, Liu PP, et al 2010a The microRNA156 and microRNA172 gene regulation cascades at post-germinative stages in *Arabidopsis*. Seed Science Research 20, 79–87.

[CIT0039] Martin RC, Asahina M, Liu PP, et al 2010b The regulation of post-germinative transition from the cotyledon- to vegetative-leaf stages by microRNA-targeted *SQUAMOSA PROMOTER-BINDING PROTEIN LIKE13* in *Arabidopsis*. Seed Science Research 20, 89–96.

[CIT0040] Murashige T, Tucker DPH 1969 Growth factor requirement of citrus tissue cultures. Proceedings of the First International Citrus Symposium Riverside, CA: University of California, 1155–1161.

[CIT0041] Nicholas KB, Nicholas HB Jr, Deerfield DW II 1997 GeneDoc: Analysis and visualization of genetic variation. Embnet News 4, 14.

[CIT0042] Nodine MD, Bartel DP 2010 MicroRNAs prevent precocious gene expression and enable pattern formation during plant embryogenesis. Genes & Development 24, 2678–2692. 21123653 10.1101/gad.1986710 PMC2994041

[CIT0043] Palovaara J, Hallberg H, Stasolla C, Hakman I 2010 Comparative expression pattern analysis of *WUSCHEL-related homeobox 2* (*WOX2*) and *WOX8*/9 in developing seeds and somatic embryos of the gymnosperm *Picea abies*. New Phytologist 188, 122–135. 20561212 10.1111/j.1469-8137.2010.03336.x

[CIT0044] Pan Z, Guan R, Zhu S, Deng X 2009 Proteomic analysis of somatic embryogenesis in Valencia sweet orange (*Citrus sinensis* Osbeck). Plant Cell Reports 28, 281–289. 18989674 10.1007/s00299-008-0633-7

[CIT0045] Pertea M, Kim D, Pertea GM, Leek JT, Salzberg SL 2016 Transcript-level expression analysis of RNA-seq experiments with HISAT, StringTie and Ballgown. Nature Protocols 11, 1650–1667. 27560171 10.1038/nprot.2016.095 PMC5032908

[CIT0046] Pulianmackal AJ, Kareem AV, Durgaprasad K, Trivedi ZB, Prasad K 2014 Competence and regulatory interactions during regeneration in plants. Frontiers in Plant Science 5, 142. 24782880 10.3389/fpls.2014.00142 PMC3990048

[CIT0047] Rhoades MW, Reinhart BJ, Lim LP, Burge CB, Bartel B, Bartel DP 2002 Prediction of plant microRNA targets. Cell 110, 513–520. 12202040 10.1016/s0092-8674(02)00863-2

[CIT0048] Robinson MD, McCarthy DJ, Smyth GK 2010 edgeR: a Bioconductor package for differential expression analysis of digital gene expression data. Bioinformatics 26, 139–140. 19910308 10.1093/bioinformatics/btp616 PMC2796818

[CIT0049] Rubio-Somoza I, Zhou CM, Confraria A, Martinho C, von Born P, Baena-Gonzalez E, Wang JW, Weigel D 2014 Temporal control of leaf complexity by miRNA-regulated licensing of protein complexes. Current Biology 24, 2714–2719. 25448000 10.1016/j.cub.2014.09.058

[CIT0050] Rupps A, Raschke J, Rümmler M, Linke B, Zoglauer K 2016 Identification of putative homologs of *Larix decidua* to *BABYBOOM* (*BBM*), *LEAFY COTYLEDON1* (*LEC1*), *WUSCHEL-related HOMEOBOX2* (*WOX2*) and *SOMATIC EMBRYOGENESIS RECEPTOR-like KINASE* (*SERK*) during somatic embryogenesis. Planta 243, 473–488. 26476718 10.1007/s00425-015-2409-y

[CIT0051] Serivichyaswat P, Ryu HS, Kim W, Kim S, Chung KS, Kim JJ, Ahn JH 2015 Expression of the floral repressor miRNA156 is positively regulated by the AGAMOUS-like proteins AGL15 and AGL18. Molecules and Cells 38, 259–266. 25666346 10.14348/molcells.2015.2311 PMC4363726

[CIT0052] Shalom L, Shlizerman L, Zur N, Doron-Faigenboim A, Blumwald E, Sadka A 2015 Molecular characterization of SQUAMOSA PROMOTER BINDING PROTEIN-LIKE (SPL) gene family from *Citrus* and the effect of fruit load on their expression. Frontiers in Plant Science 6, 389. 26074947 10.3389/fpls.2015.00389 PMC4443640

[CIT0053] Si L, Chen J, Huang X, et al 2016 OsSPL13 controls grain size in cultivated rice. Nature Genetics 48, 447–456. 26950093 10.1038/ng.3518

[CIT0054] Silva GFF, Silva EM, Guivin MAC, Ramiro DA, Figueiredo CR, Carrer H, Peres LEP, Nogueira FTS 2014 microRNA156-targeted SPL/SBP box transcription factors regulate tomato ovary and fruit development. The Plant Journal 78, 604–618. 24580734 10.1111/tpj.12493

[CIT0055] Smertenko A, Bozhkov PV 2014 Somatic embryogenesis: life and death processes during apical–basal patterning. Journal of Experimental Botany 65, 1343–1360. 24622953 10.1093/jxb/eru005

[CIT0056] Steward F, Mapes MO, Mears K 1958 Growth and organized development of cultured cells. II. Organization in cultures grown from freely suspended cells. American Journal of Botany 45, 705–708.

[CIT0057] Sun C, Zhao Q, Liu DD, You CX, Hao YJ 2013 Ectopic expression of the apple *Md-miRNA156h* gene regulates flower and fruit development in *Arabidopsis*. Plant Cell, Tissue and Organ Culture 112, 343–351.

[CIT0058] Suzuki M, McCarty DR 2008 Functional symmetry of the B3 network controlling seed development. Current Opinion in Plant Biology 11, 548–553. 18691932 10.1016/j.pbi.2008.06.015

[CIT0059] Szyrajew K, Bielewicz D, Dolata J, Wójcik AM, Nowak K, Szczygieł-Sommer A, Szweykowska-Kulinska Z, Jarmolowski A, Gaj MD 2017 MicroRNAs are intensively regulated during induction of somatic embryogenesis in *Arabidopsis*. Frontiers in Plant Science 8, 18. 28167951 10.3389/fpls.2017.00018 PMC5253390

[CIT0060] Tamura K, Stecher G, Peterson D, Filipski A, Kumar S 2013 MEGA6: molecular evolutionary genetics analysis version 6.0. Molecular Biology and Evolution 30, 2725–2729. 24132122 10.1093/molbev/mst197 PMC3840312

[CIT0061] Thakare D, Tang W, Hill K, Perry SE 2008 The MADS-domain transcriptional regulator AGAMOUS-LIKE15 promotes somatic embryo development in *Arabidopsis* and soybean. Plant Physiology 146, 1663–1672. 18305206 10.1104/pp.108.115832 PMC2287341

[CIT0062] Thompson JD, Gibson TJ, Higgins DG 2002 Multiple sequence alignment using ClustalW and ClustalX. Current Protocols in Bioinformatics Chapter 2, Unit 2.3. 10.1002/0471250953.bi0203s00 18792934

[CIT0063] Tsai AY, Gazzarrini S 2012 AKIN10 and FUSCA3 interact to control lateral organ development and phase transitions in Arabidopsis. The Plant Journal 69, 809–821. 22026387 10.1111/j.1365-313X.2011.04832.x

[CIT0064] Varkonyi-Gasic E, Wu R, Wood M, Walton EF, Hellens RP 2007 Protocol: a highly sensitive RT-PCR method for detection and quantification of microRNAs. Plant Methods 3, 12. 17931426 10.1186/1746-4811-3-12 PMC2225395

[CIT0065] Verdeil JL, Alemanno L, Niemenak N, Tranbarger TJ 2007 Pluripotent versus totipotent plant stem cells: dependence versus autonomy?Trends in Plant Science 12, 245–252. 17499544 10.1016/j.tplants.2007.04.002

[CIT0066] Wang H, Wang H 2015 The miR156/SPL module, a regulatory hub and versatile toolbox, gears up crops for enhanced agronomic traits. Molecular Plant 8, 677–688. 25617719 10.1016/j.molp.2015.01.008

[CIT0067] Wang JW 2014 Regulation of flowering time by the miR156-mediated age pathway. Journal of Experimental Botany 65, 4723–4730. 24958896 10.1093/jxb/eru246

[CIT0068] Wang JW, Czech B, Weigel D 2009 miR156-regulated SPL transcription factors define an endogenous flowering pathway in *Arabidopsis thaliana*. Cell 138, 738–749. 19703399 10.1016/j.cell.2009.06.014

[CIT0069] Wang JW, Park MY, Wang LJ, Koo Y, Chen XY, Weigel D, Poethig RS 2011 miRNA control of vegetative phase change in trees. PLoS Genetics 7, e1002012. 21383862 10.1371/journal.pgen.1002012 PMC3044678

[CIT0070] Wang L, Liu N, Wang T, Li J, Wen T, Yang X, Lindsey K, Zhang X 2018 The GhmiR157a/GhSPL10 regulatory module controls initial cellular dedifferentiation and callus proliferation in cotton by modulating ethylene-mediated flavonoid biosynthesis. Journal of Experimental Botany 69, 1081–1093. 29253187 10.1093/jxb/erx475 PMC6018973

[CIT0071] Wang X, Xu Y, Zhang S, et al 2017 Genomic analyses of primitive, wild and cultivated citrus provide insights into asexual reproduction. Nature Genetics 49, 765–772. 28394353 10.1038/ng.3839

[CIT0072] Willmann MR, Mehalick AJ, Packer RL, Jenik PD 2011 MicroRNAs regulate the timing of embryo maturation in Arabidopsis. Plant Physiology 155, 1871–1884. 21330492 10.1104/pp.110.171355 PMC3091098

[CIT0073] Wise MJ, Tunnacliffe A 2004 POPP the question: what do LEA proteins do?Trends in Plant Science 9, 13–17. 14729214 10.1016/j.tplants.2003.10.012

[CIT0074] Wu FH, Shen SC, Lee LY, Lee SH, Chan MT, Lin CS 2009 Tape-*Arabidopsis* Sandwich - a simpler *Arabidopsis* protoplast isolation method. Plant Methods 5, 16. 19930690 10.1186/1746-4811-5-16 PMC2794253

[CIT0075] Wu G, Poethig RS 2006 Temporal regulation of shoot development in *Arabidopsis thaliana* by *miR156* and its target SPL3. Development 133, 3539–3547. 16914499 10.1242/dev.02521 PMC1610107

[CIT0076] Wu XB, Wang J, Liu JH, Deng XX 2009 Involvement of polyamine biosynthesis in somatic embryogenesis of Valencia sweet orange (*Citrus sinensis*) induced by glycerol. Journal of Plant Physiology 166, 52–62. 18448195 10.1016/j.jplph.2008.02.005

[CIT0077] Wu XM, Kou SJ, Liu YL, Fang YN, Xu Q, Guo WW 2015 Genomewide analysis of small RNAs in nonembryogenic and embryogenic tissues of citrus: microRNA- and siRNA-mediated transcript cleavage involved in somatic embryogenesis. Plant Biotechnology Journal 13, 383–394. 25615015 10.1111/pbi.12317

[CIT0078] Wu XM, Liu MY, Ge XX, Xu Q, Guo WW 2011 Stage and tissue-specific modulation of ten conserved miRNAs and their targets during somatic embryogenesis of Valencia sweet orange. Planta 233, 495–505. 21103993 10.1007/s00425-010-1312-9

[CIT0079] Xie K, Wu C, Xiong L 2006 Genomic organization, differential expression, and interaction of SQUAMOSA promoter-binding-like transcription factors and microRNA156 in rice. Plant Physiology 142, 280–293. 16861571 10.1104/pp.106.084475 PMC1557610

[CIT0080] Xie M, Zhang S, Yu B 2015 microRNA biogenesis, degradation and activity in plants. Cellular and Molecular Life Sciences 72, 87–99. 25209320 10.1007/s00018-014-1728-7 PMC11113746

[CIT0081] Xing S, Salinas M, Höhmann S, Berndtgen R, Huijser P 2010 miR156-targeted and nontargeted SBP-box transcription factors act in concert to secure male fertility in *Arabidopsis*. The Plant Cell 22, 3935–3950. 21177480 10.1105/tpc.110.079343 PMC3027167

[CIT0082] Xu M, Hu T, Zhao J, Park MY, Earley KW, Wu G, Yang L, Poethig RS 2016 Developmental functions of miR156-regulated *SQUAMOSA PROMOTER BINDING PROTEIN-LIKE* (*SPL*) genes in *Arabidopsis thaliana*. PLoS Genetics 12, e1006263. 27541584 10.1371/journal.pgen.1006263 PMC4991793

[CIT0083] Xue W, Xing Y, Weng X, et al 2008 Natural variation in *Ghd7* is an important regulator of heading date and yield potential in rice. Nature Genetics 40, 761–767. 18454147 10.1038/ng.143

[CIT0084] Yamaguchi A, Wu MF, Yang L, Wu G, Poethig RS, Wagner D 2009 The microRNA-regulated SBP-Box transcription factor SPL3 is a direct upstream activator of *LEAFY*, *FRUITFULL*, and *APETALA1*. Developmental Cell 17, 268–278. 19686687 10.1016/j.devcel.2009.06.007 PMC2908246

[CIT0085] Yang X, Wang L, Yuan D, Lindsey K, Zhang X 2013 Small RNA and degradome sequencing reveal complex miRNA regulation during cotton somatic embryogenesis. Journal of Experimental Botany 64, 1521–1536. 23382553 10.1093/jxb/ert013 PMC3617824

[CIT0086] Yu N, Niu QW, Ng KH, Chua NH 2015a The role of miR156/SPLs modules in Arabidopsis lateral root development. The Plant Journal 83, 673–685. 26096676 10.1111/tpj.12919

[CIT0087] Yu ZX, Wang LJ, Zhao B, Shan CM, Zhang YH, Chen DF, Chen XY 2015b Progressive regulation of sesquiterpene biosynthesis in *Arabidopsis* and Patchouli (*Pogostemon cablin*) by the miR156-targeted SPL transcription factors. Molecular Plant 8, 98–110. 25578275 10.1016/j.molp.2014.11.002

[CIT0088] Zavattieri MA, Frederico AM, Lima M, Sabino R, Arnholdt-Schmitt B 2010 Induction of somatic embryogenesis as an example of stress-related plant reactions. Electronic Journal of Biotechnology 13, 1–9.

[CIT0089] Zhang J, Zhang S, Han S, Wu T, Li X, Li W, Qi L 2012 Genome-wide identification of microRNAs in larch and stage-specific modulation of 11 conserved microRNAs and their targets during somatic embryogenesis. Planta 236, 647–657. 22526500 10.1007/s00425-012-1643-9

[CIT0090] Zhang TQ, Lian H, Tang H, et al 2015 An intrinsic microRNA timer regulates progressive decline in shoot regenerative capacity in plants. The Plant Cell 27, 349–360. 25649435 10.1105/tpc.114.135186 PMC4456919

[CIT0091] Zhang TQ, Lian H, Zhou CM, Xu L, Jiao Y, Wang JW 2017 A two-step model for *de novo* activation of *WUSCHEL* during plant shoot regeneration. The Plant Cell 29, 1073–1087. 28389585 10.1105/tpc.16.00863 PMC5466026

[CIT0092] Zheng Q, Perry SE 2014 Alterations in the transcriptome of soybean in response to enhanced somatic embryogenesis promoted by orthologs of Agamous-like15 and Agamous-like18. Plant Physiology 164, 1365–1377. 24481137 10.1104/pp.113.234062 PMC3938626

[CIT0093] Zheng Q, Zheng Y, Ji H, Burnie W, Perry SE 2016 Gene regulation by the AGL15 transcription factor reveals hormone interactions in somatic embryogenesis. Plant Physiology 172, 2374–2387. 27794101 10.1104/pp.16.00564 PMC5129705

[CIT0094] Zheng Q, Zheng Y, Perry SE 2013 AGAMOUS-Like15 promotes somatic embryogenesis in *Arabidopsis* and soybean in part by the control of ethylene biosynthesis and response. Plant Physiology 161, 2113–2127. 23457229 10.1104/pp.113.216275 PMC3613480

