# Corrigendum: miR156-*SPL* modules regulate induction of somatic embryogenesis in citrus callus

**DOI:** 10.1093/jxb/ery197

**Published:** 2018-06-09

**Authors:** Jian-Mei Long, Chao-Yang Liu, Meng-Qi Feng, Yun Liu, Xiao-Meng Wu, Wen-Wu Guo

**Affiliations:** Key Laboratory of Horticultural Plant Biology (Ministry of Education), Huazhong Agricultural University, Wuhan, China

*Journal of Experimental Botany,* Advance Access publication: 05 April 2018, doi: 10.1093/jxb/ery132

The original online version of this paper contained an incomplete reference, omitting the name of a lead author. The reference has been updated to read as follows:

Silva GFF, Silva EM, Azevedo MDS, Guivin MAC, Ramiro DA, Figueiredo CR, Carrer H, Peres LEP, Nogueira FTS. 2014. microRNA156-targeted SPL/SBP box transcription factors regulate tomato ovary and fruit development. The Plant Journal 78, 604–618.

