# Supplementary material for: miR156-SPL modules regulate induction of somatic embryogenesis in citrus callus
Source: J Exp Bot. 2018 Apr 5;69(12):2979–93. doi: 10.1093/jxb/ery132 (PMC5972587; doi:10.1093/jxb/ery132)
Supplement: Supplementary Tables and Figuers [file ery132_suppl_supplementary-tables.pdf]

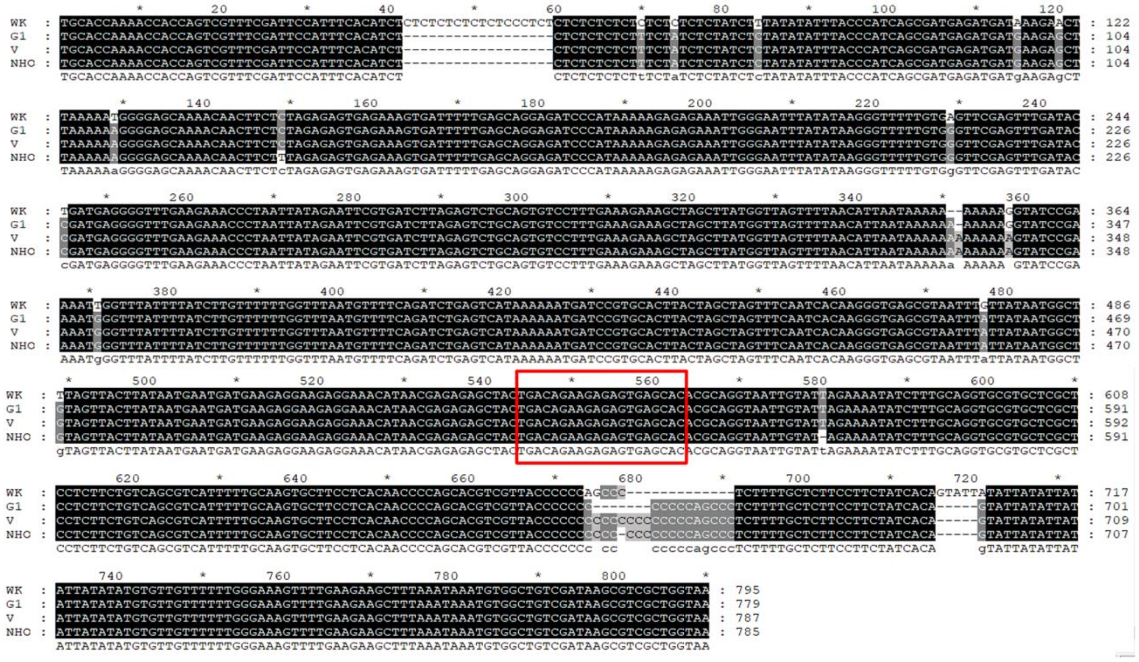

Figure S1 DNA sequence alignment of csi-miR156a gene *CsMIR156A* in different citrus genotypes with various SE capacity. The red box indicated mature csi-miR156a sequence. WK: wild kumquat; G1: ‘Guoqing No.1’ Satsuma mandarin; V: ‘Valencia’ sweet orange; NHO: ‘Newhall’ navel orange. Comparison of somatic embryogenesis (SE) capability was as follows: G1 (recalcitrant to SE) <WK<NH<V (strong SE capability).

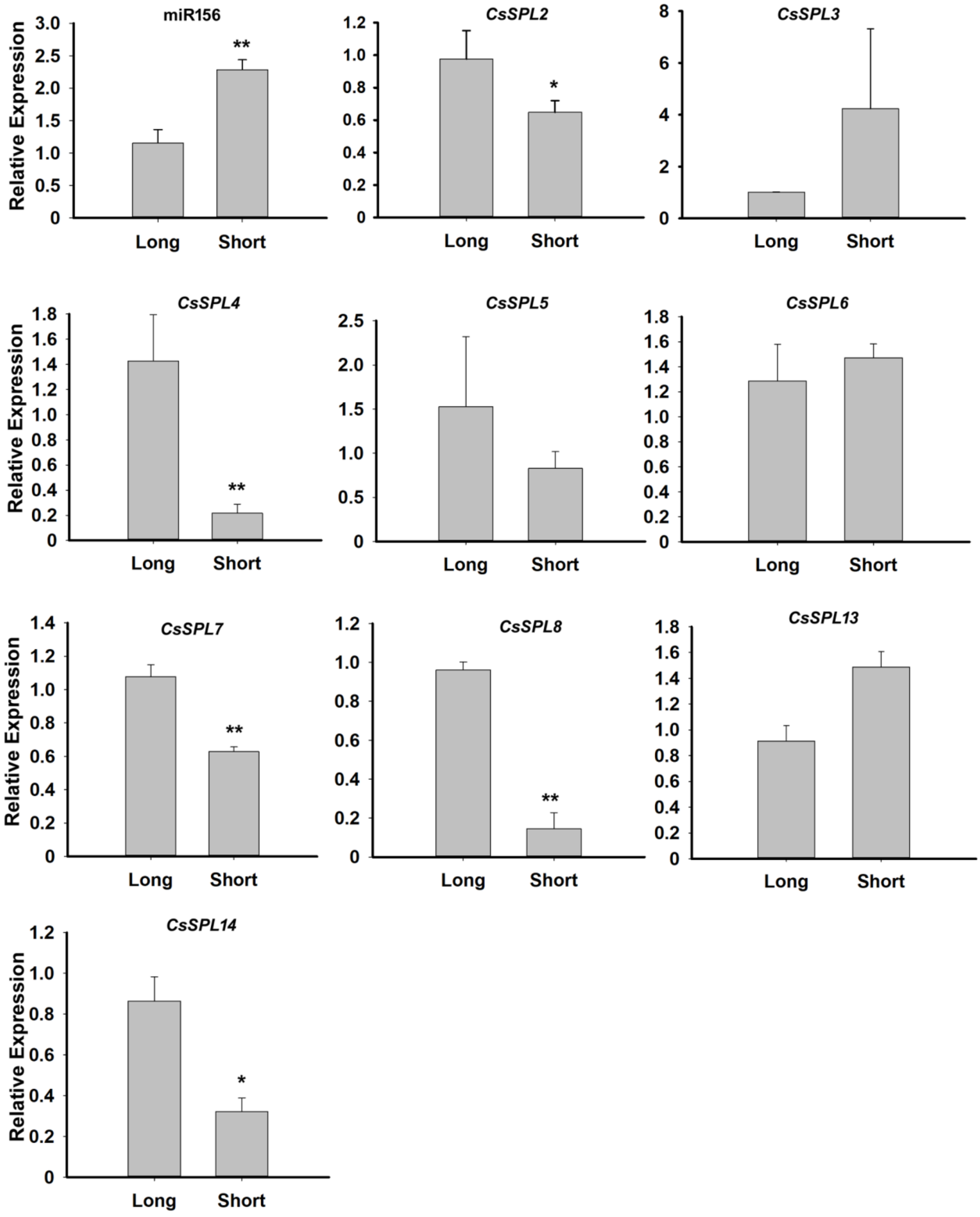

Figure S2 Expression levels of csi-miR156 and *CsSPL*s in long- and short-term preserved callus. The MT subcultured (without SE induction) WK (wild kumquat) callus was used. Error bars indicated standard deviation (SD) of three biological replicates, each containing at least three technical replicates. *CsUBL5* was used as the internal reference. Statistical significance was determined by t-test, * $P<0.05$, ** $P<0.01$.

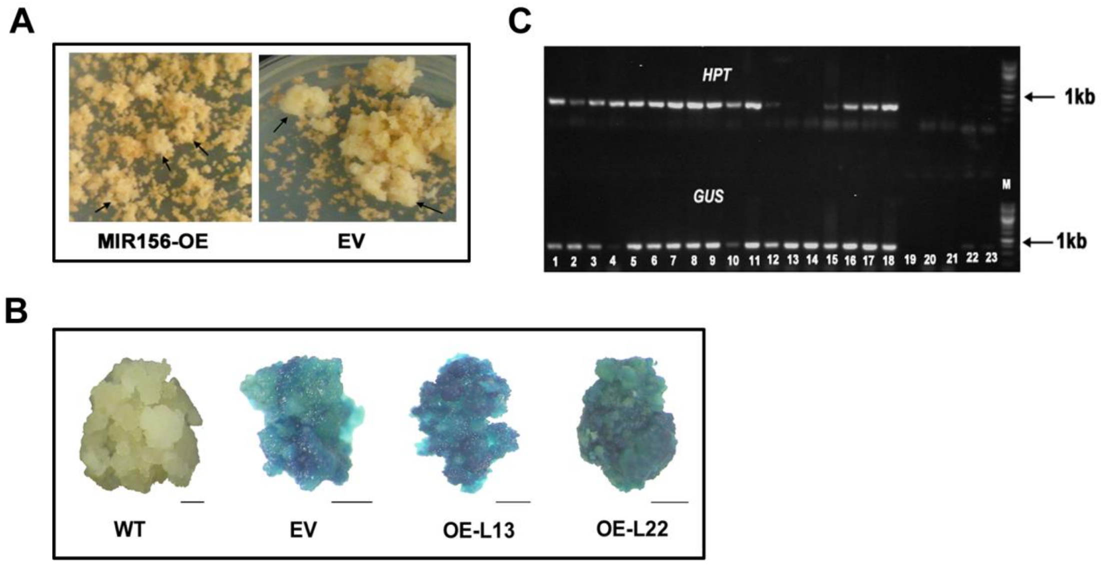

Figure S3 Generation and characterization of *MIR156a* precursor transgenic callus lines. **(**A) Generation of transformed callus. MIR156-OE: *MIR156a* transgenic callus, EV: empty vector transformed callus. The arrows indicated newly generated callus in selected medium. (B) GUS staining of transformed callus; WT: wild type, EV: empty vector, OE-L13&OE-L22: *MIR156a* transformed callus. Bar=1mm. C: Determination of positively transformed callus using PCR. The fragments of hygromycin B phosphotransferase (*HPT*) and β-glucuronidase (*GUS*) gene were amplified, respectively. 1-14: different *MIR156a* transformed lines, 15-18: empty vector control lines; 19-20: wild type; 21-23: H_2O as template control. *HPT*: *hygromycin B phosphotransferase* gene; *GUS*: *β-Glucuronidase* gene.

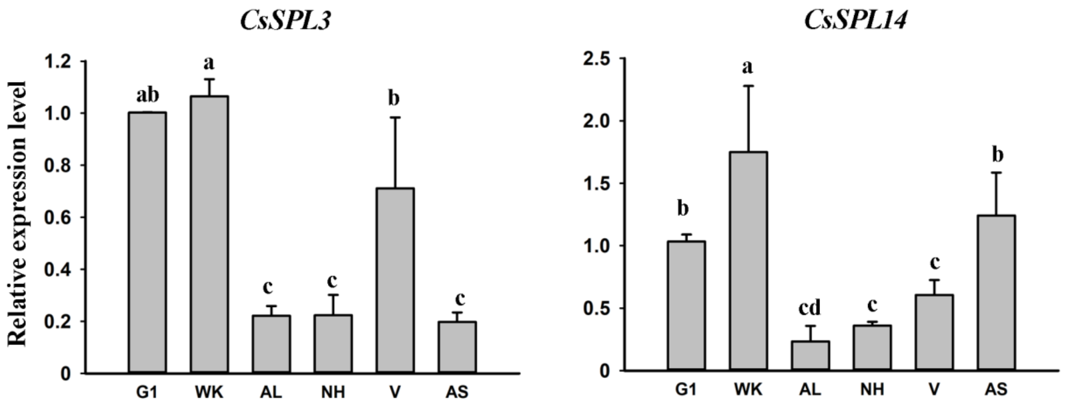

Figure S4 Expression patterns of *CsSPL3* and *CsSPL14* in callus of different citrus genotypes. Comparison of somatic embryogenesis (SE) capability was as follows: G1 (recalcitrant to SE) <WK<AL, NH<V, AS (strong SE capability). The callus without SE induction was used. *CsUBL5* was use as endogenous control. Error bars indicate SD of three biological replicates. Statistical significance was determined by multiple comparisons.

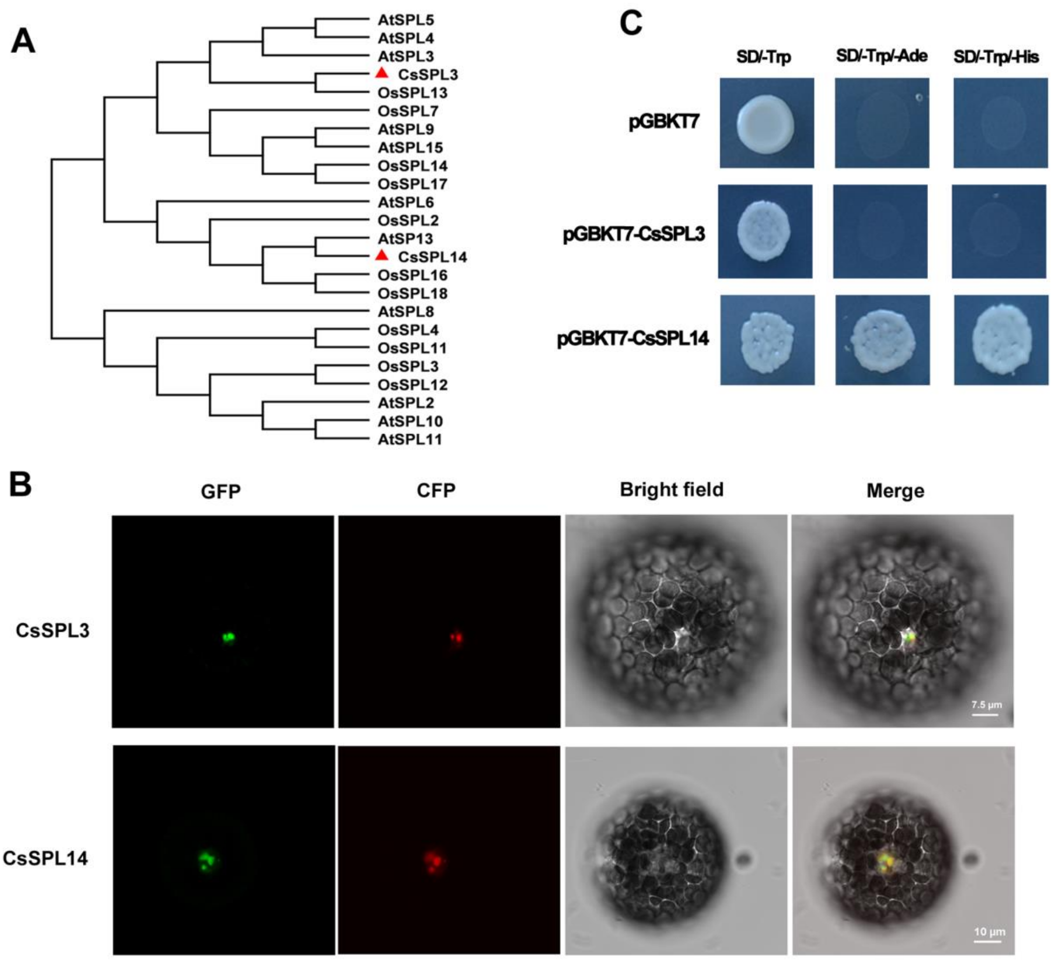

Figure S5 Phylogenetic tree, subcellular localization and transactivation activity of CsSPL3 and CsSPL14. (A) Phylogenetic analysis of CsSPL3 and CsSPL14. The protein sequences of miR156-targeted SPLs in Arabidopsis and rice were retrieved, and phylogenetic tree was generated by MEGA6. (B) Subcellular localization of CsSPL3 and CsSPL14 in Arabidopsis protoplast. CsSPL3-GFP and CsSPL14-GFP were co-transformed with OsGhd7-CFP, a nuclear marker, into Arabidopsis mesophyll protoplast, respectively. The photographs were captured under green and cyan fluorescence, bright light and merged. (C) Transactivation activity of CsSPL3 and CsSPL14 in yeast. Full length CsSPL3 and CsSPL14 were fused with the yeast GAL4 DNA-binding domain in pGBKT7 vector. The fused pGBKT7 vectors and empty vector were transformed into the yeast strain Y187, and the transactivation activity was indicated according to the yeast growth on selective medium of SD (synthetic drop-out)/-Trp, SD/-Trp/-His and SD/-Trp/-Ade.

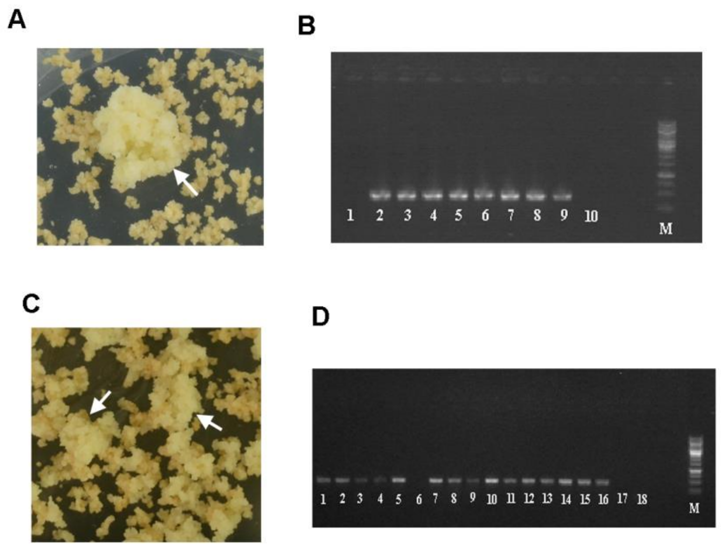

Figure S6 Generation of *CsSPL3* and *CsSPL14* RNAi calli lines. (A) Generation of callus transformed with *CsSPL3* RNAi construct. The arrows indicated newly generated callus in selected medium. (B) Determination of *CsSPL3* RNAi positively transformed callus by PCR; The fragment of aminoglycoside phosphotransferase (kanamycin-resistance gene) was amplified. 1: WT; 2-9: transgenic lines; 10: H_2O as template control. (C) Generation of callus transformed with *CsSPL14* RNAi construct. The arrows indicated newly generated callus in selected medium. (D) Determination of *CsSPL14* RNAi positively transformed callus by PCR. The fragment of aminoglycoside phosphotransferase (kanamycin-resistance gene) was amplified. 1-16: transgenic lines; 17: WT; 18: H_2O as template control. The arrows in A and C indicated regenerated callus in selected medium.

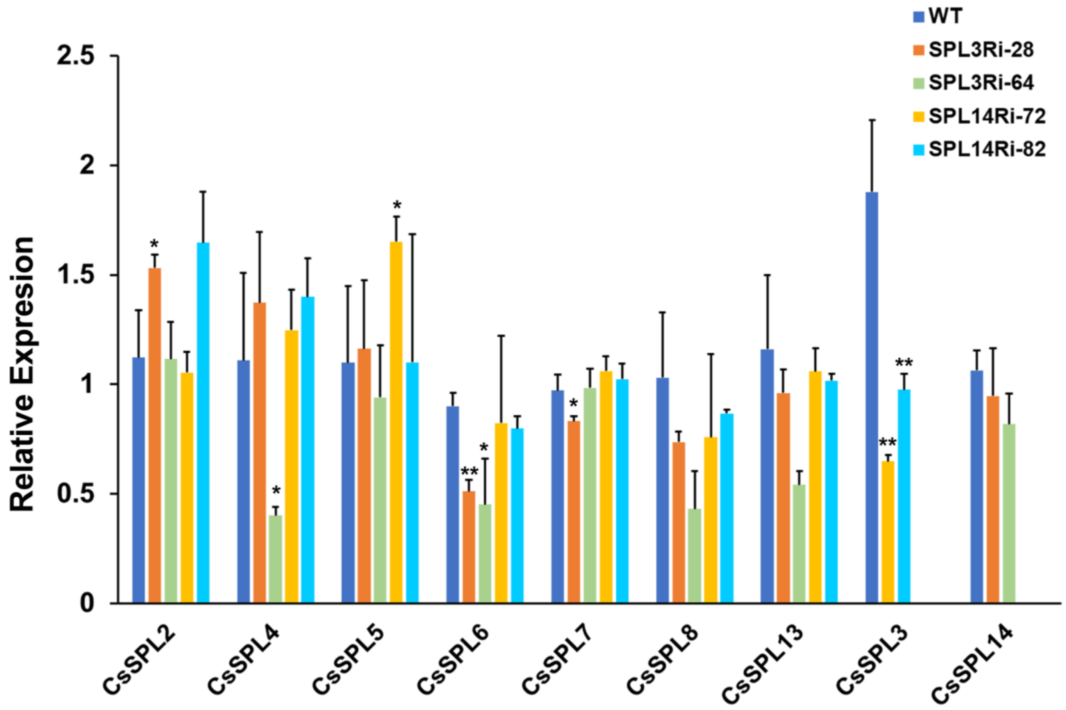

Figure S7 Transcript abundance detection of predicted miR156-targeted *CsSPL*s in *CsSPL3* and *CsSPL14* RNAi lines. The callus cultured in MT medium (without SE induction) were used for qRT-PCR. SPL3Ri-L28, SPL3Ri-L64: two *CsSPL3* RNAi lines; SPL14Ri-L72, SPL14Ri-L82: two *CsSPL14* RNAi line. Error bars indicated standard deviation (SD) of two biological replicates, each containing at least three technical replicates. *CsUBL5* was used as the internal reference. Statistical significance was determined by *t*-test, * $P<0.05$, ** $P<0.01$.

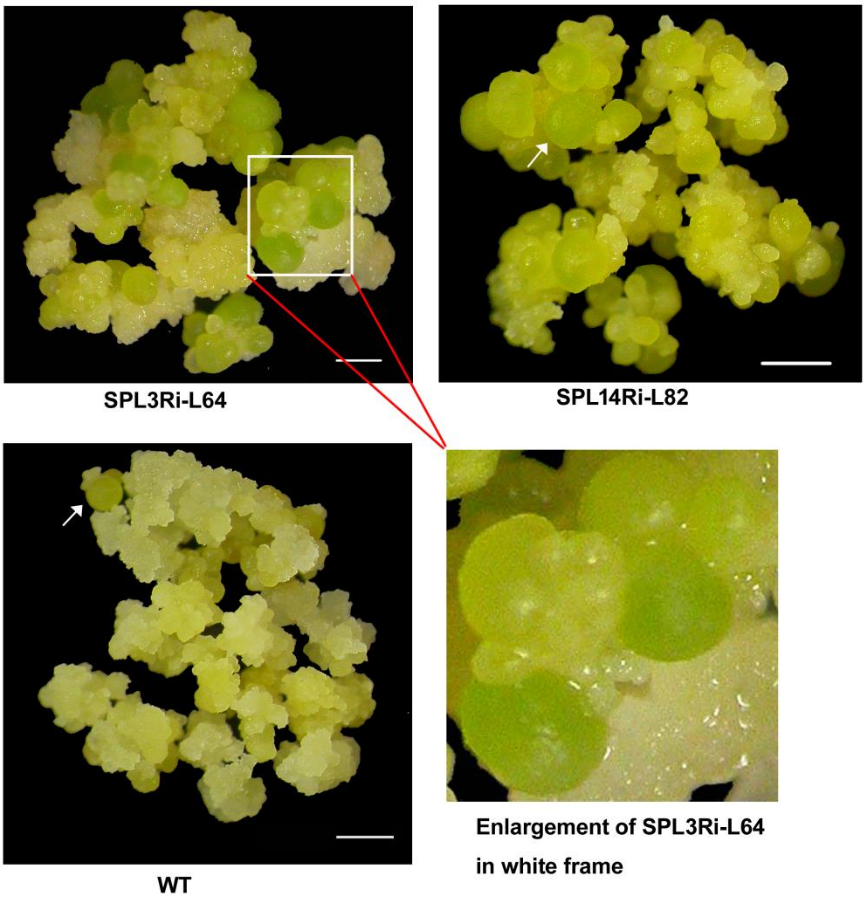

Figure S8 Somatic embryos formed in RNAi lines at 100 DAI (days after SE induction). SPL3Ri-L64: *CsSPL3* RNAi line; SPL14Ri-L82: *CsSPL14* RNAi line. Bar=1mm. The arrows indicate somatic embryos.

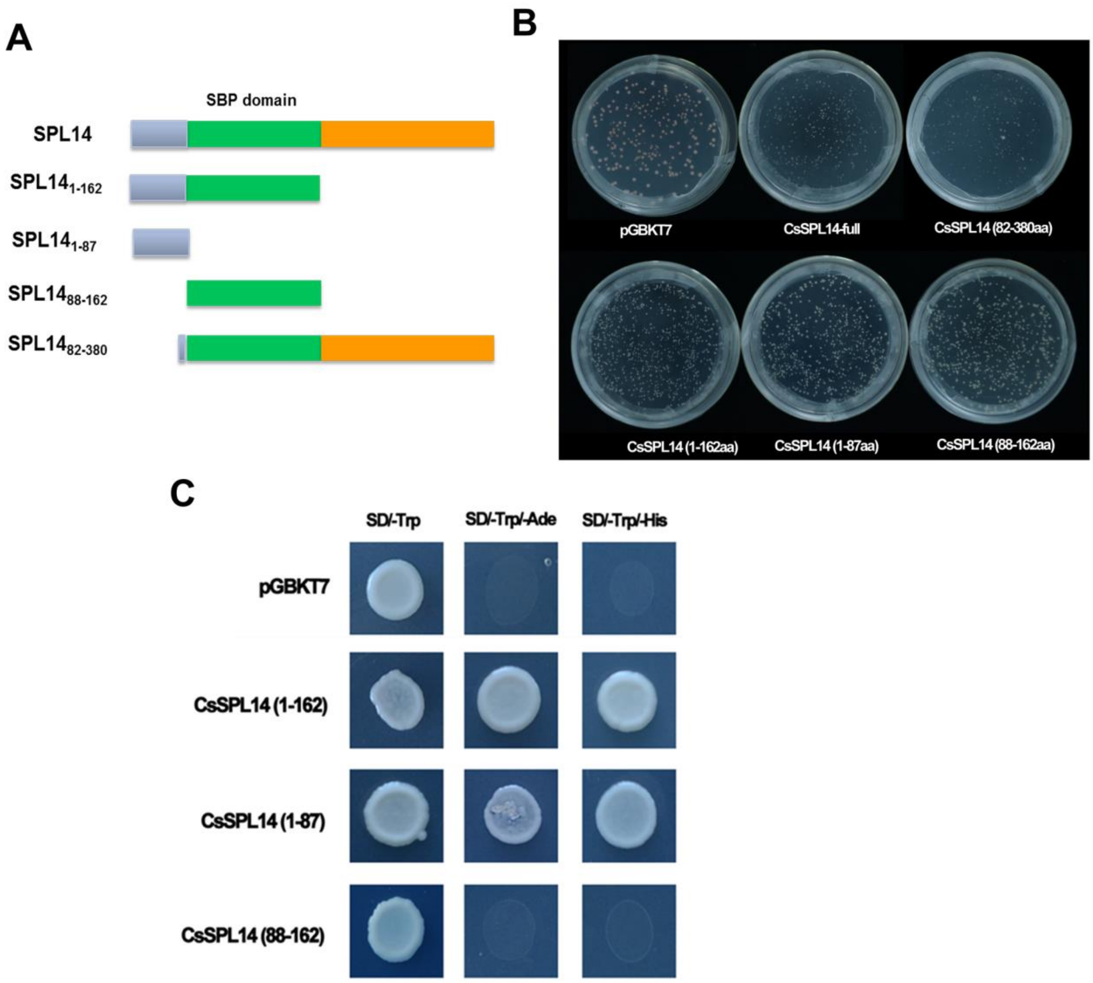

Figure S9 Yeast growth and self-activation test of truncated CsSPL14. (A) The sketch map of the truncated fragments of CsSPL14; (B) The growth of transformed yeast with different constructs. (C) Self-activation test of truncated CsSPL14. Because of the toxicity to yeast, bacteria solution of CsSPL14 (82-380aa) transformation was not obtained, thus self-activation detection was cancelled.

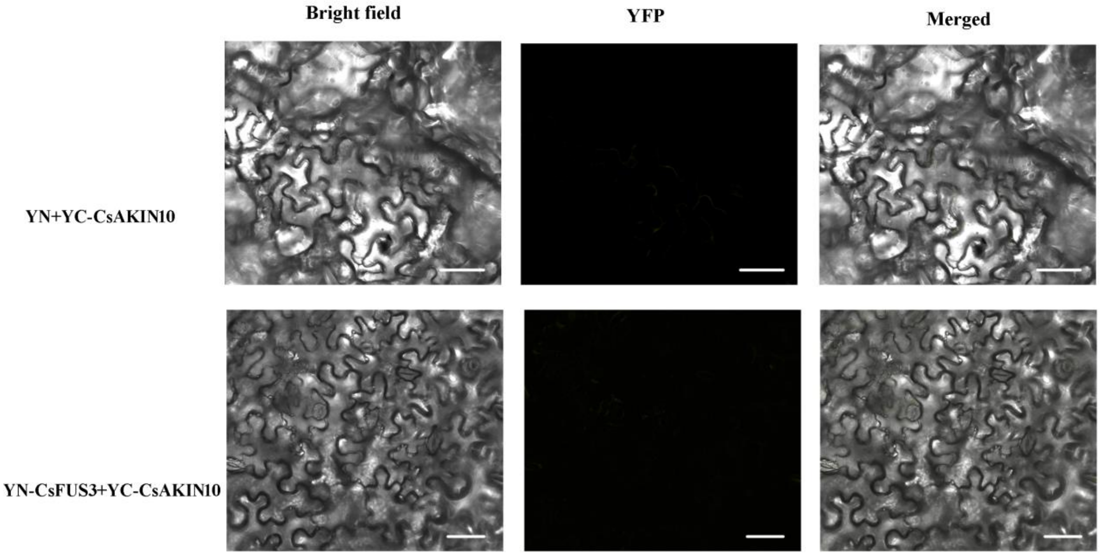

Figure S10 No interaction was detected between CsAKIN10 and CsFUS3 by BiFC. The construct of CsFUS3 fused with N terminal of YFP construct was co-infiltrated with CsAKIN10 that fused with C terminal of YFP into tobacco leaves. The co-infiltrated leaves were photographed after 3 days. Bar=50 μm.

Table S1 Primers used in this study

Primer name	sequence（5'-3'）
**Primers for RACE**	
GeneRacer-5-outer	CGACTGGAGCACGAGGACACTGA
MIR156a-5-Outer	GGAGCGAGCACGCACCTGCAAAGAT
GeneRacer-5-Inner	GGACACTGACATGGACTGAAGGAGTA
MIR156a-5-Inner	ACCTGCGTGTGCTCACTCTCTTCTGTCA
GeneRacer-3-outer	GCTGTCAACGATACGCTACGTAACG
MIR156a-3-Outer	AGAAGAGAGTGAGCACACGCAGGTA
GeneRacer-3-Inner	CGCTACGTAACGGCATGACAGTG
MIR156a-3-Inner	TCTTTGCAGGTGCGTGCTCGCTCCT
MIR156a-F	TGCACCAAAACCACCAGTCGT
MIR156a-R	TTACCAGCGACGCTTATCGACA
**Primers for miR156a targets *SPL*s RNAi constructs**	
CsSPL3-attb1	AAAAAGCAGGCTAAGTGCTTCTTCAGTAAGGTGCC
CsSPL3-attb2	AGAAAGCTGGGTGGTAATAACGAGTGTTCCCTCAA
CsSPL14-attb1	AAAAAGCAGGCTGCCAGTGATACCCCTCAAACA
CsSPL14-attb2	AGAAAGCTGGGTGGTGCTTGCCATAAGCATTTA
attB1-adapter	GGGGACAAGTTTGTACAAAAAAGCAGGCT
attB2-adapter	GGGGACCACTTTGTACAAGAAAGCTGGGT
**Primers for transactivation assay of two SPLs**	
PBD-SPL3-F	ATATGGCCATGGAGGCCGAATTCATGGACAACGATATGGAAGA
PBD-SPL3-R	GGGGTTATGCTAGTTATGCGGCCGCCTACTGAGGACCTACCCCTC
PBD-SPL14-F	ATATGGCCATGGAGGCCGAATTCATGGACTGGAACTTGAAACC
PBD-SPL14-R	GGGGTTATGCTAGTTATGCGGCCGCCTACTCCCAATGAAAGGGA
**primers for truncated CsSPL14 bait vectors**	
PBD-SPL14-H1-R	GGGGTTATGCTAGTTATGCGGCCGCCTAAGGTTTCCTTCGTCTTCTGTTG
PBD-SPL14-H2-R	GGGGTTATGCTAGTTATGCGGCCGCCTATGACGCAGTCTGGCTTCC
PBD-SPL14-Q1-F	ATATGGCCATGGAGGCCGAATTCAGCCAGACTGCGTCATGTC

PBD-SBP-domain-F	ATATGGCCATGGAGGCCGAATTCTGTCTTGTTGATGGGTGTGA
PBD-SBP-domain-R	GGGGTTATGCTAGTTATGCGGCCGCCTAAGGTTTCCTTCGTCTTC
**primers for AD vectors construction of potential proteins interacted with CsSPL14**	
PAD-CsAKIN10-F	GCCATGGAGGCCAGTGAATTCATGGATGGAGCCTCTAACCG
PAD-CsAKIN10-R	TACGATTCATCTGCAGCTCGAGTTAAAGAACCCGAAGCTGTGC
**primers for BiFC assay**	
PCL112-SPL14-F	AAAAAGCAGGCTTCATGGACTGGAACTTGAAACC
PCL112-SPL14-R	AGAAAGCTGGGTGCTACTCCCAATGAAAGGGAATTG
PCL113-AKIN10-F	AAAAAGCAGGCTTCATGGATGGAGCCTCTAACCGAAG
PCL113-AKIN10-R	AGAAAGCTGGGTGTTAAAGAACCCGAAGCTGTGC
**primers for verification of RNA-seq by qRT-PCR**	
CsFUS3-qF	TTGCCGAGCGTCCTTCAC
CsFUS3-qR	AAGGGAAGAGACCAGCAGGCAG
CsPOD-qF	GGGCTTGATGATGGTTGA
CsPOD-qR	GAGGGTTGTTCTCAGAAAGG
CsZFP-qF	TGTGCAATAATTCAAGACTACTCCG
CsZFP-qR	TCTCAGAAAGACGCCTTCTCTC
CsLEA6-qF	GCTCTGTTTCGGTTTCAGCAGTTCT
CsLEA6-qR	TTCAGCTCTTTCTTGTCCTGACGA
CsLEA4-qF	GGAAGAAACACAGTAGAATCCGCCA
CsLEA4-qR	TCTCCATCGGGTCCCTCGTC
CsAKIN10-qF	GTTCCAAGCCCATCTTCC
CsAKIN10-qR	AATCCCATCTTAACCACTTCC

Table S2 Summary of RNA-seq data

Sample	raw reads	clean reads	aligned reads	mapped rate
WT-G40-BR1	14,204,258	13601584	12148935	89.32%
WT-G40-BR2	13,556,650	12974625	11561688	89.11%
WT-G60-BR1	15,856,574	15203851	13543590	89.08%
WT-G60-BR2	16,642,318	15910971	14195768	89.22%
OE-G40-BR1	14,769,896	14112290	12495022	88.54%
OE-G40-BR2	15,952,460	15257508	13478483	88.34%
OE-G60-BR1	15,506,111	14992660	13362958	89.13%
OE-G60-BR2	16,552,734	15971150	14219115	89.03%

WT, wild type; OE, miR156 overexpression lines; G40, 40 DAI; G60, 60 DAI; BR1/BR2 indicated two biological replicates respectively.

Table S3 Correlation of coefficients between biological replicates of RNA-seq data

Samples	WT-G40-BR1	WT-G40-BR2	OE-G40-BR1	OE-G40-BR2	WT-G60-BR1	WT-G60-BR2	OE-G60-BR1	OE-G60-BR2
WT-G40-BR1	1.00	**0.98**	0.54	0.52	0.96	0.96	0.81	0.78
WT-G40-BR2		1.00	0.56	0.53	0.96	0.96	0.81	0.75
OE-G40-BR1			1.00	**0.94**	0.44	0.44	0.82	0.86
OE-G40-BR2				1.00	0.41	0.42	0.82	0.91
WT-G60-BR1					1.00	**0.98**	0.75	0.67
WT-G60-BR2						1.00	0.76	0.68
OE-G60-BR1							1.00	**0.95**
OE-G60-BR2								1.00

WT, wild type; OE, csi-miR156a overexpression lines; G40, 40 DAI; G60, 60 DAI; BR1/BR2 indicated two biological replicates respectively.
